# Mycorrhizal feedbacks influence global forest structure and diversity

**DOI:** 10.1038/s42003-023-05410-z

**Published:** 2023-10-19

**Authors:** Camille S. Delavaux, Joseph A. LaManna, Jonathan A. Myers, Richard P. Phillips, Salomón Aguilar, David Allen, Alfonso Alonso, Kristina J. Anderson-Teixeira, Matthew E. Baker, Jennifer L. Baltzer, Pulchérie Bissiengou, Mariana Bonfim, Norman A. Bourg, Warren Y. Brockelman, David F. R. P. Burslem, Li-Wan Chang, Yang Chen, Jyh-Min Chiang, Chengjin Chu, Keith Clay, Susan Cordell, Mary Cortese, Jan den Ouden, Christopher Dick, Sisira Ediriweera, Erle C. Ellis, Anna Feistner, Amy L. Freestone, Thomas Giambelluca, Christian P. Giardina, Gregory S. Gilbert, Fangliang He, Jan Holík, Robert W. Howe, Walter Huaraca Huasca, Stephen P. Hubbell, Faith Inman, Patrick A. Jansen, Daniel J. Johnson, Kamil Kral, Andrew J. Larson, Creighton M. Litton, James A. Lutz, Yadvinder Malhi, Krista McGuire, Sean M. McMahon, William J. McShea, Hervé Memiaghe, Anuttara Nathalang, Natalia Norden, Vojtech Novotny, Michael J. O’Brien, David A. Orwig, Rebecca Ostertag, Geoffrey G. (‘Jess’) Parker, Rolando Pérez, Glen Reynolds, Sabrina E. Russo, Lawren Sack, Pavel Šamonil, I-Fang Sun, Mark E. Swanson, Jill Thompson, Maria Uriarte, John Vandermeer, Xihua Wang, Ian Ware, George D. Weiblen, Amy Wolf, Shu-Hui Wu, Jess K. Zimmerman, Thomas Lauber, Daniel S. Maynard, Thomas W. Crowther, Colin Averill

**Affiliations:** 1 ETH Zurich, Department of Environmental Systems Science, Zurich, Switzerland; 2 Department of Biological Sciences, Marquette University, Milwaukee, WI USA; 3 Department of Biology, Washington University in St. Louis, St. Louis, MO USA; 4 Department of Biology, Indiana University, Bloomington, IN USA; 5 Forest Global Earth Observatory, Smithsonian Tropical Research Institute, Panama City, Panama; 6 Department of Biology, Middlebury College, Middlebury, VT USA; 7 Center for Conservation and Sustainability, Smithsonian Conservation Biology Institute, National Zoological Park, Washington, DC USA; 8 Forest Global Earth Observatory, Smithsonian Conservation Biology Institute, National Zoological Park, Washington, DC USA; 9 Geography & Environmental Systems, University of Maryland, Baltimore County, Baltimore, MD USA; 10 Biology Department, Wilfrid Laurier University, Waterloo, Canada; 11 Herbier National du Gabon, Institut de Pharmacopée et de Médecine Traditionelle, Libreville, Gabon; 12 Department of Biology, Temple Ambler Field Station, Temple University, Ambler, PA USA; 13 Conservation Ecology Center, Smithsonian Conservation Biology Institute, National Zoological Park, Washington, DC USA; 14 National Biobank of Thailand, National Science and Technology Development Agency, Khlong Nueng, Pathum Thani Thailand; 15 School of Biological Sciences, University of Aberdeen, Aberdeen, UK; 16 Taiwan Forestry Research Institute, Taipei City, Taipei, Taiwan, ROC; 17 State Key Laboratory of Biocontrol, School of Ecology/School of Life Sciences, Sun Yat-sen University, Guangzhou, China; 18 Department of Life Science, Tunghai University, Taichung City, Taiwan, ROC; 19 School of Life Sciences, Sun Yat-sen University, Guangzhou, China; 20 Department of Ecology and Evolutionary Biology, Tulane University, New Orleans, LA USA; 21 Institute of Pacific Islands Forestry, USDA Forest Service, Hilo, HI USA; 22 Department of Environmental Sciences, Wageningen University, Wageningen, The Netherlands; 23 Department of Ecology and Evolutionary Biology, University of Michigan, Ann Arbor, MI USA; 24 Department of Science and Technology, Uva Wellassa University, Badulla, Sri Lanka; 25 Gabon Biodiversity Program, Center for Conservation and Sustainability, Smithsonian National Zoo and Conservation Biology Institute, Gamba, Gabon; 26 University of Hawaii at Manoa, 1910 East-West Rd., Honolulu, HI USA; 27 Water Resources Research Center, University of Hawaii at Manoa, Honolulu, USA; 28 Environmental Studies Department, University of California, Santa Cruz, Santa Cruz, CA USA; 29 Department of Renewable Resources, University of Alberta, Edmonton, Canada; 30 Department of Forest Ecology, Silva Tarouca Research Institute, Průhonice, Czech Republic; 31 Department of Natural and Applied Sciences, University of Wisconsin-Green Bay, Green Bay, WI USA; 32 Environmental Change Institute, School of Geography and the Environment, University of Oxford, Oxford, UK; 33 Department of Ecology and Evolutionary Biology, University of California, Los Angeles, Los Angeles, CA USA; 34 Department of Biology, University of Hawaii, Hilo, HI USA; 35 School of Forest Resources and Conservation, University of Florida, Gainesville, FL USA; 36 School of Forest, Fisheries, and Geomatics Sciences, University of Florida, Gainesville, USA; 37 Department of Forest Management, W. A. Franke College of Forestry and Conservation, University of Montana, Missoula, MT USA; 38 The Wilderness Institute, W. A. Franke College of Forestry and Conservation, University of Montana, Missoula, MT USA; 39 Department of Natural Resources and Environmental Management, University of Hawaii at Manoa, Honolulu, USA; 40 The Ecology Center, Utah State University, Logan, UT USA; 41 Wildland Resources Department, Utah State University, Logan, UT USA; 42 Department of Biology, University of Oregon, Eugene, OR USA; 43 Forest Global Earth Observatory, Smithsonian Environmental Research Center, Edgewater, NJ USA; 44 Centre National de la Recherche Scientifique et Technologique, Ouagadougou, Burkina Faso; 45 Programa Ciencias de la Biodiversidad, Instituto de Investigacion de Recursos Biologicos Alexander von Humboldt, Bogota, Colombia; 46 Biology Centre, Institute of Entomology, Czech Academy of Sciences, Budějovice, Czech Republic; 47 Estación Experimental de Zonas Áridas, Consejo Superior de Investigaciones Científicas, Almería, Spain; 48 Harvard Forest, Harvard University, Petersham, MA USA; 49 Forest Ecology Group, Smithsonian Environmental Research Center, Edgewater, NJ USA; 50 The Royal Society SEARRP (UK/Malaysia), Kota Kinabalu, Sabah Malaysia; 51 School of Biological Sciences and Center for Plant Science Innovation, University of Nebraska – Lincoln, Lincoln, NE USA; 52 Department of Natural Resources and Environmental Studies, National Dong Hwa University, Hsinchu, Taiwan, ROC; 53 School of the Environment, Washington State University, Pullman, WA USA; 54 UK Centre for Ecology & Hydrology, Bailrigg, UK; 55 Department of Ecology, Evolution, and Environmental Biology, Columbia University, New York, NY USA; 56 Tiantong National Forest Ecosystem Observation and Research Station, School of Ecological and Environmental Sciences, East China Normal University, Shanghai, China; 57 U.S. Forest Service, Institute of Pacific Islands Forestry, Pacific Southwest Research Station, Hilo, HI USA; 58 Department of Plant & Microbial Biology, University of Minnesota, St. Paul, MN USA; 59 Botanical Garden Division, Taiwan Forestry Research Institute, Taipei City, Taiwan, ROC; 60 Department of Environmental Sciences, University of Puerto Rico, Rio Piedras, Puerto Rico

**Keywords:** Biodiversity, Biogeography, Forest ecology

## Abstract

One mechanism proposed to explain high species diversity in tropical systems is strong negative conspecific density dependence (CDD), which reduces recruitment of juveniles in proximity to conspecific adult plants. Although evidence shows that plant-specific soil pathogens can drive negative CDD, trees also form key mutualisms with mycorrhizal fungi, which may counteract these effects. Across 43 large-scale forest plots worldwide, we tested whether ectomycorrhizal tree species exhibit weaker negative CDD than arbuscular mycorrhizal tree species. We further tested for conmycorrhizal density dependence (CMDD) to test for benefit from shared mutualists. We found that the strength of CDD varies systematically with mycorrhizal type, with ectomycorrhizal tree species exhibiting higher sapling densities with increasing adult densities than arbuscular mycorrhizal tree species. Moreover, we found evidence of positive CMDD for tree species of both mycorrhizal types. Collectively, these findings indicate that mycorrhizal interactions likely play a foundational role in global forest diversity patterns and structure.

## Abstract (web summary)

A global study suggests that mycorrhizal fungi play a key role in determining forest diversity patterns and community structure, with mycorrhizal tree type systematically mediating the strength of conspecific negative density dependence.

## Introduction

The global latitudinal plant species diversity gradient is one of the most striking biogeographical patterns on Earth, with plant diversity highest in warm, moist tropical regions^1,2^. This pattern has inspired a wealth of ecological and evolutionary hypotheses to explain the mechanisms that structure forest communities across latitudes. The Janzen-Connell hypothesis^3,4^ posits that the accumulation of species-specific enemies near an adult tree reduces the recruitment of conspecific individuals. This localized reduction in conspecific recruitment—known as negative conspecific density dependence (CDD)—creates a patchwork of available spaces for heterospecific species, and may ultimately support higher tree species diversity in a forest. Negative CDD has been shown to be stronger at lower latitudes, and in wetter^5^, warmer^6^ sites, which has the potential to maintain the higher plant diversity in these regions^7,8^. Since the Janzen-Connell hypothesis was first proposed, the role of soil-borne pathogens in driving CDD has received experimental and theoretical support^9–12^. However, plant mutualists, such as mycorrhizal fungi, also have the potential to influence plant recruitment in the opposite direction. By increasing the survival of conspecifics^10,13,14^, these mutualists might also mediate CDD^15–18^, with the potential to counteract negative pathogen-driven feedbacks^19,20^.

Mycorrhizal fungi are critical plant mutualists that shape the structure of forest communities worldwide^21–23^. These fungi have formed mutualisms with plants for at least 450 million years^24^, with the fungus providing vital nutritional resources in return for photosynthetically-derived carbon from the plant host^25^. These mycorrhizal fungi could counteract negative enemy-mediated CDD by promoting a local build-up of species-specific mutualists around adult trees, resulting in increased recruitment and performance of conspecific saplings. A growing body of evidence suggests that differences between the two major mycorrhizal plant types^25^—arbuscular mycorrhizal (AM) and ectomycorrhizal (EM)—may mediate the strength of these mycorrhizal effects with consequences for global forest diversity patterns. Specifically, EM plant species may experience weaker negative CDD than AM plant species for to two major reasons. First, EM plant species exhibit relatively greater host-plant specificity, or affinity^9,10,17,26^, which would lead to greater recruitment or performance of conspecifics in areas wither higher conspecific density. Second, EM fungi are thought to confer greater pathogen protection relative to AM fungi, particularly via physical root protection^27^ (but see^28^). These differences between mycorrhizal types would lead to the expectation that EM plant species are able to counteract enemy-driven negative CDD to a greater extent than AM plant species, ultimately experiencing weaker negative CDD. Although local and regional studies have found patterns in line with this hypothesis^27,29–31^, it is unknown whether or not these patterns emerge at the global scale. A pattern of weaker negative CDD among EM trees, coupled with the well-known mycorrhizal latitudinal gradient of increasingly EM-dominated forests towards higher latitudes^23,32–34^, would provide a complementary mechanism to explain the shift from stronger to weaker CDD with increasing latitude, which could influence the latitudinal gradient in tree species diversity.

The global latitudinal diversity gradient exists in conjunction with a second prominent biogeographical pattern: bimodality of forest community composition with respect to mycorrhizal type. Most forests on Earth are composed predominantly of either AM or EM tree species^33,35^. In fact, Connell hypothesized that strong positive feedbacks within EM forests may explain the existence of EM-dominated forests in otherwise diverse tropical latitudes^36,37^. If EM tree species can survive in tropical systems, what prevents EM tree species from invading into AM-dominated forests and mixing with AM trees? On the contrary, if low latitude, AM-dominated communities have strong negative CDD that supports greater diversity, this negative CDD should facilitate community membership of both AM and EM trees. One explanation for why AM-dominated forests exclude EM trees is that AM tree species have higher recruitment when surrounded by heterospecific tree species with the same mycorrhizal type, or positive conmycorrhizal density dependence^19,38^ (CMDD). Positive CMDD may be greater for AM tree species due to their relatively lower host specificity^25,39^, which should allow AM tree species to more readily interact with and benefit from mycorrhizal fungi associated with other AM tree species (conmycorrhizal heterospecifics). In contrast, EM tree species may not benefit substantially from other EM tree species due to the relatively higher specificity of their plant-fungal mutualism, potentially preventing them from taking advantage of EM fungi associated with heterospecific neighbors to the same extent as AM tree species. The presence of positive CMDD would predict that monomycorrhizal ecosystems, or ecosystems dominated by one mycorrhizal type, should be disproportionately common compared to mixed mycorrhizal forests^40^, and suggest mycorrhizal compatibility may act as a fundamental filter on forest community membership at a global scale.

**Fig. 1:**
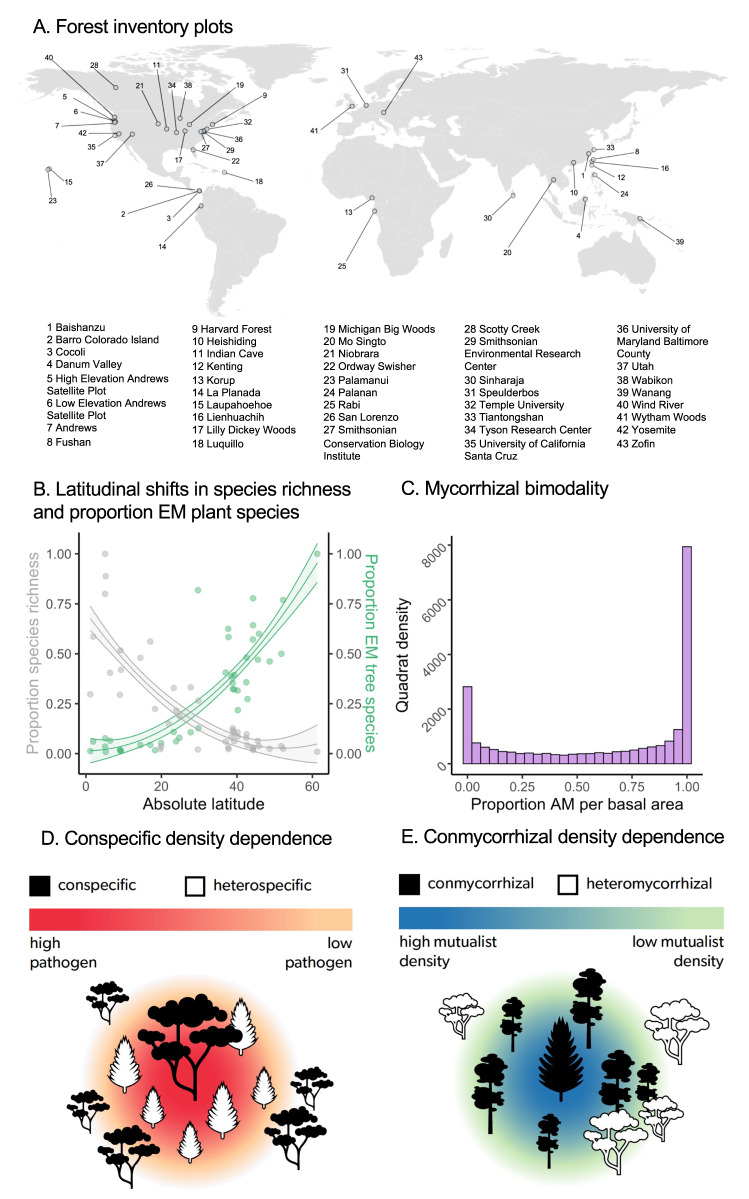
Geographical extent of dataset captures known biogeographical patterns. A map of the 43 ForestGEO network sites included in the study (**A**), collectively comprising over 3 million stems. Major expected relationships including the latitudinal gradient in tree species diversity (scaled to the maximum species richness across sites; gray, *p* < 0.001) and the gradient from AM dominated to EM-dominated sites (proportion of EM tree species in a site; green, *p* = 0.02) with increasing latitude (**B**) as well as the mycorrhizal bimodality of forests (**C**; proportion of AM per basal area of all tree species) are captured by our data. Conceptual figures depicting negative conspecific density dependence (CDD) in recruitment due to hypothesized higher densities of species-specific enemies around a conspecific adult tree (**D**) and positive conmycorrhizal density dependence (CMDD) in recruitment due to hypothesized shared mutualists with an adult tree of a conmycorrhizal heterospecific species (**E**).

Here, we test for systematic differences in spatial patterns of per-capita sapling density associated with CDD as a function of mycorrhizal type, as well as the presence of positive community level, within mycorrhizal type feedbacks (CMDD) for each mycorrhizal type. We hypothesize that EM tree species show a higher density of conspecific saplings per adult (per-capita sapling density), as predicted under weaker negative CDD (H_1_). This would be consistent with greater pathogen protection and/or specificity between mutualist and host within EM tree species, which should alleviate the effect of enemy-mediated negative CDD to a stronger degree than for AM tree species. We further expect that AM tree species will show greater per-capita sapling density around heterospecific AM trees, indicative of positive community level mycorrhizal feedback, or CMDD (H_2_). This would be consistent with lower host specificity of AM tree species, allowing them to take greater advantage of mycorrhizal fungi associated with surrounding AM heterospecific species. To test these hypotheses, we use global forest inventory data generated from 43 large-scale research sites, leveraging data collected as part of the Forest Global Earth Observatory (ForestGEO) Network^41,42^. These data encompass measurements across boreal, temperate and tropical ecosystems, including 2,775,129 trees and 4161 tree species, spanning almost 70° of latitude (Fig. 1A). This massive dataset allows us to test – for the first time – the generality of mycorrhizal mediation of density dependence and plant diversity at a global scale. The sites also capture the two major gradients underlying our hypotheses: the latitudinal gradient in tree species diversity (Fig. 1B) and the mycorrhizal bimodality of forest systems (Fig. 1C). We evaluate spatial predictions of CDD (Fig. 1D) and CMDD (Fig. 1E), using statistical analyses and null models that address many previous criticisms, by both (1) fitting individual species-by-site level models and (2) fitting a single global integrated model that allows us to predict the per-capita sapling density for each mycorrhizal type and species. Collectively, these analyses allow us to ask to what extent *positive* interactions with mycorrhizal fungi mediate forest structure and diversity, with implications for plant biogeography and the maintenance of forest biodiversity worldwide.

## Results and discussion

### Mycorrhizal mediation of conspecific density dependence may contribute to the latitudinal species diversity gradient

**Fig. 2:**
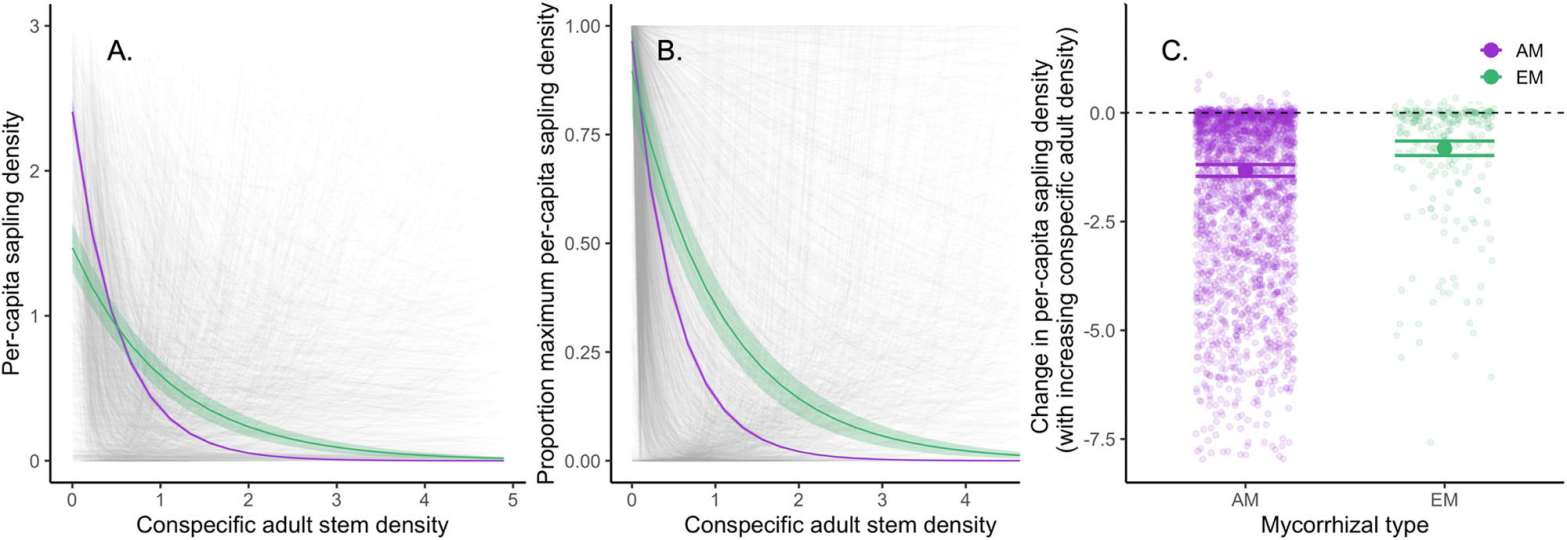
Mycorrhizal type mediates strength of conspecific density dependence. Negative conspecific density dependence (CDD), estimated as the degree to which sapling densities decrease with increasing conspecific adult tree density (per-capita sapling density), is consistently stronger for arbuscular mycorrhizal (AM) compared to ectomycorrhizal (EM) tree species. The three panels show results from the integrated global model that incorporates random slopes and intercepts for each tree species-by-site, using raw sapling densities (**A**), sapling densities scaled to the maximum sapling density of each mycorrhizal type (**B**), and species-by-site estimates of change in per-capita sapling density with a standard increase in conspecific adult density (1 conspecific adult) extracted from this model (**C**, *n* = 2469, *p* < 0.001). Gray lines in (**A**) and (**B**) represent species-by-site curves; AM and EM tree species are shown in purple and green, respectively. Solid circles and horizontal lines in (**C**) represent means estimated from the model and standard errors, respectively.

Here we show that EM tree species have weaker negative CDD than AM tree species (H_1_), consistent with forest mycorrhizal type playing a key role in structuring global patterns of tree species diversity. As predicted, the density of conspecific saplings per adult (hereafter per-capita sapling density) decreased less with increasing conspecific adult density for EM compared to AM tree species (Fig. 2, Table S2, and S3). We find this pattern using both a single global integrated model (Fig. 2A and B) and individual species-by-site level models (Fig. 2C). In addition, this pattern holds across temperate and tropical biomes (biome by mycorrhizal type interaction *p* = 0.17) and is consistent across sites (Fig. S1). These patterns are consistent with the hypothesis (H_1_) that EM fungi counteract enemy-driven negative CDD to a greater extent than AM fungi, likely due to either greater specificity^26^ of the EM plant-fungal mutualism or greater pathogen protection conferred by EM fungi to their hosts^27^. Our results align with previous studies examining associations between mycorrhizal type and CDD at local scales or within biomes^29,30^. However, our findings show that this pattern holds at the globe scale, as our results provide a much broader-scale test of this hypothesis, including 43 sites across 70° of latitude, representing over 3 million stems and nearly 4000 tree species, and using multiple modeling approaches. As such, our findings provide strong support for the idea that mycorrhizal types mediate the strength of CDD across forests worldwide.

Observed differences in per-capita sapling density between mycorrhizal types are consistent with mycorrhizal fungi influencing forest diversity broadly and the latitudinal gradient in species diversity. Specifically, the latitudinal gradient from AM-dominated tropics to EM-dominated temperate and boreal regions (Fig. 1B), coupled with a consistent difference in CDD between the mycorrhizal types (Fig. 2), suggests a novel mechanism that could explain the latitudinal shift in tree CDD^43^ (Fig. S2). Both the strengthening of negative CDD with decreasing absolute latitude^7^ and the latitudinal diversity gradient itself^5^ have previously been hypothesized to be influenced by greater pathogen pressure or host-specificity in warmer, moist tropical latitudes^5^. However, another complementary mechanism is that the greater proportion of EM tree species^44^ may explain the weakening of negative CDD and overall forest diversity declines, with increasing latitude (Fig. S3). However, many additional factors are known to influence the latitudinal diversity gradient^45^, and the extent to which these patterns in local (plot-level alpha) diversity scale up to the regional (gamma) diversity reflected in this gradient remains unresolved^46^. Moving forward, an integrated understanding of both plant enemies, especially soil-borne pathogens, and fungal mutualists, will be fundamental to our understanding of forces structuring forest diversity and composition. Future work should address the relative contribution of these two important functional groups of microbes that may impact recruitment, growth, and survival in opposing directions. It will also be important to continue to expand experimental tests of different plant-mycorrhizal benefits^28^ and how they vary across species, populations and habitats^47,48^ to deepen our understanding of how mycorrhizal relationships mediate plant diversity.

### Positive conmycorrhizal density dependence may reinforce mycorrhizal bimodality of forests

**Fig. 3:**
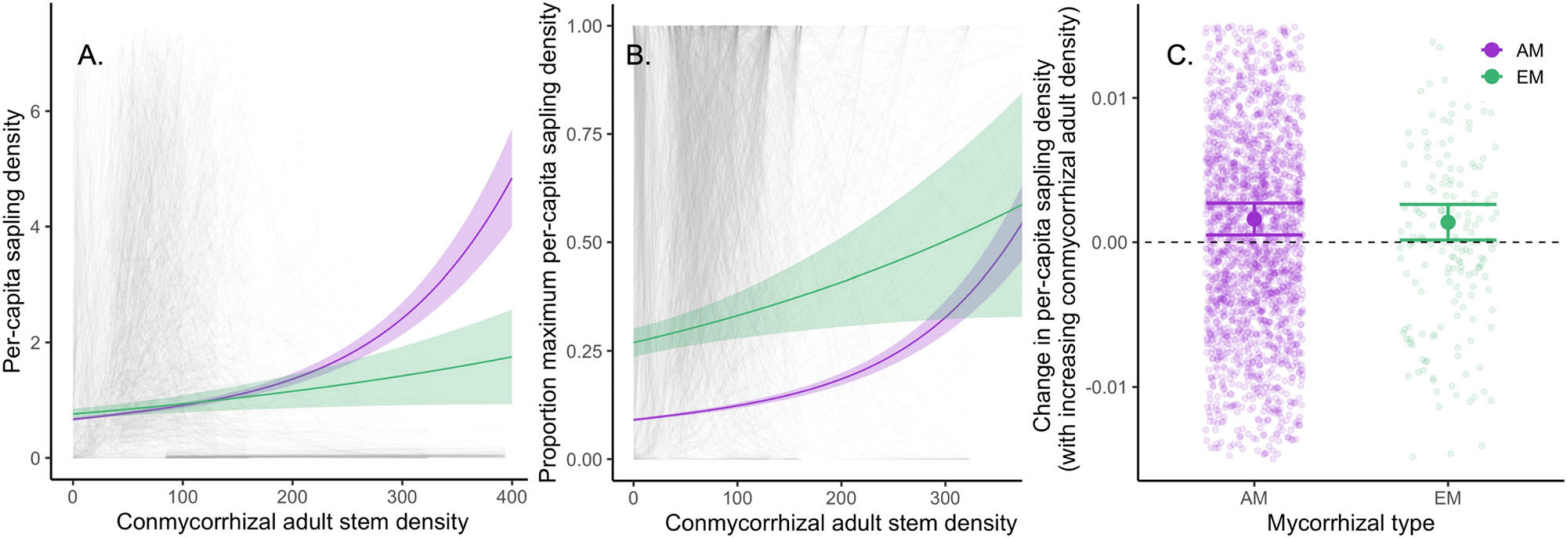
AM tree species benefit from shared mycorrhizal fungi. AM tree species experience positive conmycorrhizal density dependence (CMDD), measured as conmycorrhizal per-capita sapling density, while EM plant species show weaker evidence for positive CMDD. Positive CMDD can be seen for both mycorrhizal types in the global model (**A**, **B**) and for AM tree species in the species-by-site estimates of change in per-capita sapling density with a standard increase in conmycorrhizal adult tree density (1 conmycorrhizal adult) extracted from this model (**C**, *n* = 2428). Gray lines in (**A**) and (**B**) represent species-by-site curves; AM and EM tree species are shown in purple and green, respectively. Solid circles and horizontal lines in (**C**) represent means estimated from the model and standard errors, respectively.

Our results support the hypothesis that AM tree species on average experience positive CMDD (H_2,_ Fig. 3, Table S4). As predicted, per-capita sapling density of AM tree species increased with increasing densities of other AM (conmycorrhizal heterospecific) tree species (hereafter conmycorrhizal per-capita sapling density). Across all species at all sites, conmycorrhizal per-capita sapling density of AM tree species was significantly greater than zero (*p* < 0.001; Fig. 3C). These results are consistent with the low specificity of AM plant species allowing these species to benefit from mycorrhizal mutualists associated with other AM plants species, and in effect share fungal mutualists across plant species^23,33,49^. EM tree species also showed a positive conmycorrhizal per-capita sapling density in the global model (Fig. 3A and B). When using species-by-site estimates derived from this model, EM tree species also exhibit positive CMDD, but this response is not statistically significant (*p* > 0.05, Fig. 3C). Because the global model was influenced more by tree species that have greater representation in the data, this model is likely biased toward abundant species, or those that occur across many sites (particularly temperate areas). In contrast, when using species-by-site conmycorrhizal per-capita sapling density estimates in a subsequent model, each species is weighted equally. This may indicate that abundant tree species within the EM functional group drive this positive conmycorrhizal per-capita sapling density at the global level and why it is not significantly positive at the species level. Alternatively, greater uncertainties in CMDD estimates of rare species may result from reduced statistical power, as species were removed from sites with complete mycorrhizal dominance of one type. This may play a greater role among EM tree species which represent a much smaller number of tree species globally. The lack of strong consistent signal within EM tree species may also be due to the higher host specificity of EM trees, which may limit their ability to benefit from mycorrhizal fungi associated with other EM tree species. Further, although we find that AM tree species show more positive conmycorrhizal per-capita sapling density than EM tree species when accounting for site variation, these patterns vary substantially between sites (Fig. S4). Although previously tested in glass-house studies^27,38^ and assessed regionally^19^, we show for the first time that globally, AM tree species exhibit spatial patterns consistent with positive CMDD, consistent with shared AM mutualists between these plants.

This positive community-level feedback (CMDD) in AM tree species may help explain the mycorrhizal bimodality of forests^35^, and particularly why EM tree species are not typically found in AM-dominated forests (and vice-versa). Although AM tree species experience stronger negative CDD, which could facilitate establishment of EM tree species in AM-dominated patches, the observed positive CMDD could prevent EM tree species from establishing in these patches. EM forests may also experience a positive conmycorrhizal feedback^50–52^ despite generally higher host specificity that may reflect the reduced ability of EM trees to associate with EM fungi of neighboring heterospecifics. This monomycorrhizal tendency of EM plant species and filtering out of AM plant species may also be in part due to differential nutrient economies between EM and AM systems. Specifically, EM immobilization of inorganic nitrogen may reduce nitrogen availability for AM fungi^19^ and their associated plant hosts^53^, which may maintain EM-dominated patches by preventing AM plant species from establishing. Furthermore, the ability of EM plant species to access organic phosphorus may also contribute to their dominance in certain patches^54^, especially in phosphorus limited tropical ecosystems. Further, although evidence is limited^19,55,56^, common mycorrhizal networks may play a role in maintaining mycorrhizal monodominance^57^ if tree species benefit substantially from being connected to this network. Interestingly, negative CDD seems qualitatively stronger than positive CMDD; although community level CMDD may maintain AM and EM segregation, there is still pervasive negative CDD among species in both groups. Moving forward, it will also be important to investigate drivers of community assembly in forests with mixed mycorrhizal types, such as Dipterocarp forests in Southeast Asia^58,59^. Either in isolation or combination, positive CMDD in AM and EM tree species has the potential to explain the mycorrhizal bimodality observed across forests worldwide.

Although our modeling and analytical approach addresses several previous concerns raised for using spatial analyses of data collected from a single timepoint^60,61^, future work should examine these same patterns using dynamic data incorporating growth and survival over several censes. One shortcoming common in such analyses is the unrealistic assumption of linearity between adult and sapling density. In our analyses, we have accommodated for the non-linearity of this relationship through the use of generalized additive models (GAMs). These models allow for non-linearity, without making assumptions regarding the shape of the relationship. Another widespread issue with previous approaches was a bias toward more common species^62,63^, as common species have more data for species specific models; however, this may lead to an underestimation of negative density dependence. We have overcome this by using a global integrated model, which substantially increased the data that could be retained for analyses. In our case, using the global integrated model resulted in an increase of 45% and 39% species and sites respectively (for CDD analyses) when compared to the species-by-site analyses. We recognize that other processes such as soil edaphic properties (i.e., habitat filtering), disturbance, seed dispersal distance, species herd protection, seed size^5^ and seed germination^64^ may all influence these patterns. Here, we address the impact of factors influencing these aggregating processes through use of a null model that incorporates empirically-informed dispersal limitation, clumping in the adult distribution, and adult mortality. Although there is a growing consensus that dynamic data are ideal when testing for density dependence^19^, this would have sharply decreased the number of plots we would be able to incorporate in our study. Moreover, there is also evidence that these types of analyses may be in agreement. For example, recent work has shown that across an elevational gradient in Oregon, CDD examined through long-term data on tree survival was more strongly negative at low elevations^65^; we found a similar trend when looking at the spatial signature of CDD (per-capita sapling density; median plot CDD) across three of these elevational plots included in our analyses. In addition, spatial patterns of dispersion of saplings away from conspecific adults more holistically addresses the processes hypothesized to underlie CDD (i.e., negative CDD making more space available for other species) than survival and growth. Nonetheless, it will be important to more systematically compare the single census approach to dynamic data on growth and survival, allowing an assessment of whether inferences using spatial patterns are consistent with survival and growth.

Collectively, we show that AM and EM tree species differ systematically in strength of CDD in sapling density across the globe. We suggest that this major difference in CDD among mycorrhizal types may offer a mechanism to explain variation in forest diversity and ultimately the latitudinal gradient in tree species diversity. The shift in relative abundance of mycorrhizal types from AM-dominated low latitudes to EM-dominated high latitudes, alongside consistently weaker negative CDD for EM tree species, may explain the weaker CDD and lower tree species diversity observed at higher latitudes. Further, differences in CMDD, with AM tree species showing significantly positive CMDD, may explain why highly diverse AM-dominated forests exhibiting stronger negative CDD tend not to harbor EM tree species, with a segregation of forests by mycorrhizal type resulting in the mycorrhizal bimodality of forests. AM tree species exhibit positive CMDD, potentially reinforcing their own mycorrhizal type and excluding alternative (EM) mycorrhizal types. Therefore, consistent differences in CDD and CMDD between the mycorrhizal types may contribute to both the latitudinal diversity gradient and mycorrhizal bimodality globally. Together, our study underlines the major role that mutualistic soil microbes, especially mycorrhizal fungi, may have in mediating forest dynamics and global forest diversity patterns.

## Methods

### Forest census data synthesis

Our study included tree-census data from 43 sites that are part of the ForestGEO network^6^ (Table S1; 23 temperate, 19 tropical, 1 boreal). Each site contains a large forest plot in which all free standing woody stems (hereafter trees) ≥1 cm DBH (diameter at breast height, 1.3 m from the ground) were measured, identified, and geolocated following the same protocol^41^. We converted the data from one census from each site to a universal format, using the following variables extracted from the censuses for each individual stem: full latin name, genus, species, the x-coordinate position within the plot, the y-coordinate position within the plot, the DBH, and status (alive or dead). We kept all live trees whether they were broken or otherwise damaged to retain as much recorded data as possible; all dead stems (9.9% of stems) were excluded from downstream analyses. The most recent census was used for every site except for Yosemite, where we used the earliest census (2009–10) to avoid the confounding effect of fire-caused mortality in later censuses^66^, and the Luquillo plot, where the census in 2011 prior to hurricane Maria^67^ (2016) was used. This resulted in a dataset comprised of 43 sites and a total of 3,299,000 stems (2,775,129 trees, or main stems) across 4161 species. All metadata was calculated using stem data, whereas subsequent analyses were always carried out using only main stems (the largest stem of an individual).

### Site and species metadata

We determined metadata at three levels: (1) the species level, (2) the site level and (3) the species-by-site level; all metadata was calculated using the full stem dataset. At the species level (i.e., for each species), we determined species level or if not possible, putative genera-based mycorrhizal status. To do this, we relied on the FungalRoot database^68^, first assigning to known species mycorrhizal status, and if species data was unavailable in either the census data or the FungalRoot database, to genus level mycorrhizal status. We only analyzed species that were reported as strictly AM or EM in this database, and removed all ambiguous categories (AMEM, AMNM, where NM is non-mycorrhizal), other mycorrhizal types, and unmatched plant species; this resulted in the removal of 6.1% of observations (stems). We determined the proportion of EM and AM tree species, as well as the proportion of total basal area comprised of EM trees, for each site by summarizing species level mycorrhizal data. At the site level, we extracted latitude and longitude directly from the ForestGEO website (forestgeo.si.edu), and used these geographical coordinates to extract mean annual temperature and precipitation from the CHELSA database^69^. Finally, at the species-by-site level, we used the census data combined with species level data to calculate site specific species abundance and relative abundance. All metadata was calculated using the full stem dataset.

### Overview of statistical analyses

Our analytical approach consisted of (1) modeling sapling density around nearby conspecific and heterospecific adults (per-capita sapling density, the number of saplings per quadrat divided by the number of conspecific or heterospecific adult trees per quadrat), (2) predicting the change in per-capita sapling density for a standard increase in adult density, and (3) using either models (A) directly or (B) resulting predictions in a second set of models to test our hypotheses (see sections ‘*Per-capita sapling density GAM models’* and *‘Estimating change in per capita sapling density from models’* below for details*)*. We tested for (1) systematic differences in conspecific saplings per adult (per-capita sapling density) between the two mycorrhizal types; we hypothesized that EM tree species show a higher density of conspecific saplings per adult (per-capita sapling density), as predicted under weaker negative CDD (H_1_). We also test for evidence of positive community level mycorrhizal feedback, or CMDD in both mycorrhizal types (H_2_).

Our analyses were carried out using two modeling approaches: species-by-site and global. All models were run using two datasets: one was the full dataset to estimate per-capita sapling density associated with conspecific tree density dependence (CDD), and the other was a subset of the full dataset used to estimate conmycorrhizal per-capita sapling density associated with CMDD. Where possible, we examined the predictive power of three types of adults on focal species per-capita sapling density: conspecific adults, heterospecific adults that shared the same mycorrhizal status (conmycorrhizal heterospecific adults, CMH), and heterospecific adults that did not share the same mycorrhizal status (heteromycorrhizal heterospecific adults, HMH). Because our models for CMDD required both heterospecifics that were conmycorrhizal and heteromycorrhizal, the data used in these analyses represented a subset of sites, as sites where all species were one mycorrhizal type were excluded (i.e., because CMDD cannot be estimated where all trees are of one mycorrhizal type; sites excluded: Kenting, Palamanui, and Scotty Creek). We first constructed models for each species-by-site combination (species-by-site combinations: CDD = 1189, CMDD = 1388) and then constructed one global integrated model including a random intercept and slope for each species-by-site combination. We report results from the global model in the main text as this approach retains more species and is the most parsimonious, but all results are given in the supplementary information (Tables S2 and S3). Finally, to verify the robustness of our modeling approach and minimize effects of habitat preference, dispersal, adult mortality, and other abiotic drivers, we conducted null models to account for per-capita sapling density not associated with CDD or CMDD (please see section ‘*Null models*’ for full description). In addition, we tested for phylogenetic correlation in our two metrics of CDD and CMDD, making sure that this was sufficiently low to justify using non-phylogenetically constrained models for our analyses.

### Size class delineation

We classified main stems as adults or saplings based on size class^7^. We classified saplings as stems smaller than 10 cm in DBH, with all other stems classified as adults. However, if less than 20% of stems of a particular species were classified as adults using this cutoff, the cutoff for sapling was lowered to 5 cm DBH. If this resulted in less than 20% of stems of a given species being classified as adults, the cutoff was lowered to 2 cm. No further classification was done, as 1 cm is the smallest measured stem in this database.

### Per-capita sapling density GAM models

We used GAMs in the mgcv package^70,71^ to test for the impact of conspecific adults and either (1) all heterospecific adults or (2) conmycorrhizal heterospecific adults (CMH) and heteromycorrhizal heterospecific adults (HMH) on per-capita sapling density. These two model versions relied on the two datasets for CDD or CMDD described above. Using GAM models instead of linear regression allowed us to model non-linear relationships between adult density and conspecific sapling density, an improvement upon previous methods that used linear models^7,62,63^. The unit of replication we used was the quadrat, representing 20 × 20 m subplots across each plot. We chose this unit as it is a reasonable scale at which to expect spatial patterns due to density dependence based on previous studies^72,73^. Finally, all adult density measurements use distance-weighted abundances as described in LaManna et al.^63,71^, which account for adults outside of focal quadrats; these are referred to as adult density in model descriptions below. Specifically, adults were weighted equally within focal quadrats, and those outside were assigned a decreasing weight according to a Clark 2dt dispersal kernel starting at the edge of the quadrat. Species were only included in models if saplings and adults both occurred in a minimum of ten quadrats.

In total, species-by-site CDD models included data from 29 sites and 1647 species, species-by-site CMDD models included data from 18 sites and 1318 species. For global models, CDD models included all 43 sites and 2097 species, while CMDD models included 40 sites and 2065 species. Because the global models allowed us to retain substantially more sites and species, we report the global model results and estimates generated from these models in the main text (Tables S2, S3A and S4A). However, all species-by-site model results are presented in Tables S3B and S4B.

For species-by-site CDD GAM models, we fit a GAM version of the Ricker function; that is, we predicted per-capita sapling density from adult density. Predictor variables included conspecific and heterospecific adult density. The model had the following formula:

1 $$S=A{e}^{r+{cA}+{dH}}$$

Where *S* is sapling density within a quadrat, *A* is conspecific adult density (distance-weighted adult density), *r* is the sapling density in areas with no conspecific adults, *c* is the effect of increasing conspecific adult density on *S*, *H* is heterospecific adult density, and *d* is the effect of increasing heterospecific adult density on *S*. Model family was set to negative binomial with a log link. For CMDD models, predictor variables were the same as for CDD models, except that heterospecific adult density was replaced by conmycorrhizal heterospecific and heteromycorrhizal heterospecific adult density. The model had the following formula:

2 $$S=A{e}^{r+{cA}+{{{{{\rm{dM}}}}}}+{{{{{\rm{fH}}}}}}}$$

Where *S* is sapling density within a quadrat, *A* is conspecific adult density, *r* is the sapling density in areas with no conspecific adults, *c* is the effect of increasing conspecific adult density on *S*, M is the conmycorrhizal heterospecific adult density and *H* is heteromycorrhizal heterospecific adult density, and *d* and f are the effect of increasing heterospecific conmycorrhizal or heteromycorrhizal adult density on *S*, respectively.

For the global integrated models, we used models that included all available data in one model, with each species-by-site combination as a random intercept and slope for *c*. For these models (2; CDD and CMDD), we used the BAM() function in the mgcv package^74^ as this fits a GAM to large datasets more efficiently^75^. For the CDD global model, predictor variables included conspecific and heterospecific adult density, both with a slope of mycorrhizal type (AM or EM). Including this slope of mycorrhizal type represents an interaction between conspecific adult density and mycorrhizal type, enabling a direct test of our hypothesis of whether mycorrhizal type mediates CDD. We further included a random intercept and slope of species-by-site combination for conspecific adult density; this enabled us to extract species-by-site estimates for subsequent analyses. Model family was again set to negative binomial with a log link. Finally, we reran this with the CMDD dataset; all predictor variables were the same as the model using the CDD dataset, except heterospecific adult density was split into conmycorrhizal heterospecific and heteromycorrhizal heterospecific adult density. Additionally, both conspecific adult density and conmycorrhizal heterospecific adult density included a slope of mycorrhizal type to directly test for mycorrhizal mediation of both per-capita sapling density and conmycorrhizal per-capita sapling density.

### Estimating change in per capita sapling density from models

After running these recruitment models, we calculated the per-capita sapling density in relation to conspecific and conmycorrhizal adults by comparing the change in sapling recruit density (*S*) from a standard increase in number of conspecific or conmycorrhizal adults, respectively. Next, a log ratio was calculated to determine the relative change due to the addition of one conspecific or conmycorrhizal compared to one heterospecific adult. Because our predictions incorporate both conspecific and heterospecific adult densities, our reported CDD per-capita sapling density values (or CMDD per-capita sapling density values) represent the change in per-capita sapling density relative to heterospecific density dependence.

For species-by-site GAMs, each species and model was characterized by its own range of conspecific adult densities. Because we wanted to predict change in per-capita sapling density (1) only where this was captured by the real data, and (2) for a standardized change of adult conspecific densities, we determined the window over which adding 1 conspecific adult would retain the most species as possible for the final analyses. For the change in per-capita sapling density, we iteratively tested for all possible start and end values of the addition of 1 conspecific adult to determine the optimal starting conspecific adult value. We repeated this process for 0.5 and 0.75 conspecific adults and all estimates were highly correlated (average correlation 0.996); therefore, small changes in the number of conspecifics added should not strongly influence our estimates. The final window across which we retained most species occurred between 0.067 and 1.067 conspecific adults. Values are fractions instead of whole numbers of adults because of distance weighting applied prior to analyses.

Analogous calculations were done to predict the impact of increase in conmycorrhizal adult density. However, to more realistically represent the much larger densities of conmycorrhizal compared to conspecific adults (median conmycorrhizal adult density per quadrat = 65.36). an optimal starting point for conmycorrhizal adult density was set separately. This followed the same procedure as for the conspecific starting point, where we iteratively tested for all possible start and end values of the addition of 1 conmycorrhizal adult. The final window across which we retained most species occurred between 67.275 and 68.275 conmycorrhizal adults. In addition, for CMDD models, we assigned species-specific proportions of conmycorrhizal heterospecific adult (CMH) and heteromycorrhizal heterospecific adult (HMH) densities in our predictions as proportions of the heterospecific adults density for all species. For corresponding CDD and CMDD global integrated models, we were able to retain all species and calculate the change in per-capita sapling density from 0 to 1 conspecific adults, or conmycorrhizal adults.

### Hypothesis testing using estimates

In order to test our hypotheses, we either (1) plotted global integrated model results directly or (2) used species-by-site estimates in change of per-capita sapling density or conmycorrhizal per-capita sapling density from either the species-by-site or global models to conduct generalized linear models (GLMs). To plot our global integrated model results directly (Figs. 2A, B,  3A and B), we used the predict.gam() function from the R package mgcv to predict the per-capita sapling density with increasing conspecific (or conmycorrhizal) adult densities. When predicting these curves and confidence intervals, we kept other fixed effects at their mean and excluded the influence of species-by-site random intercepts and slopes. This allowed us to visualize the overall patterns of CDD and CMDD between mycorrhizal types across all species-by-site combinations.

For models using species-by-site estimates extracted from our GAM models (either species-by-site or global models), we joined these estimates with the metadata to conduct GLMs to test our hypotheses. Using both species-by-site and global model results, we tested at the site and species-by-site level. Site level analyses allowed us to investigate broad global scale patterns of CDD and associated drivers, while species-by-site level analyses allowed us to address our overarching questions of differences in CDD and CMDD by species’ mycorrhizal type. At the site level, we conducted exploratory analyses using GLMs predicting species abundance weighted median CDD and CMDD by latitude, mean annual temperature, and mean annual precipitation (Fig. S2). We also explored relationships between each species richness and proportion of EM plant species with latitude (Fig. 1B) as well as species richness and proportion EM plant species (Fig. S3). Species abundance weighted median was calculated using species-by-site estimates, by applying the weighted.median() function in base R and weighting by log abundance (natural log) of each species within each unique site. Each model included one predictor variable (as listed above); we were unable to combine multiple predictors due to collinearity. At the species-by-site level, we tested for the impact of mycorrhizal type on CDD and CMDD, first setting site as random effect, and then as an interacting fixed effect to test a site dependent impact of mycorrhizal type on tree density dependence. Because CMDD showed no significant difference between mycorrhizal types, we tested whether the estimated mean of each mycorrhizal type was significantly different than zero using a *t* test. Finally, we also examined the relationship between tree species abundance and CDD (Fig. S5).

### Null models

To evaluate whether observed differences in our estimates of CDD and CMDD were explained by other processes that influence densities and spatial distributions of saplings and adults, we ran null models that incorporated other processes that may influence spatial signatures but are not necessarily related to CDD^63,70,71^.

These include clumping in the adult distribution (distributions of adults were fixed to reflect clumping processes unrelated to CDD, including habitat affinity), adult mortality (allowing for some proportion of adults to spawn recruits and then die), and dispersal limitation based on the empirically measured range of possible mean dispersal distances for each species (based on tree species maximum height) from recent meta-analyses of dispersal^7,63,76^. Specifically, we used a dispersal-kernel null model^62,63^ that maintained observed locations of adults to account for factors that influence adult density, and then dispersed sapling recruits using empirically estimated dispersal kernels. For each tree species, we estimated its mean dispersal distance using recent meta-analyses of interspecific relationships between mean seed dispersal distances and maximum plant height^7,47,63,76^. Adult mortality was also incorporated into this null model to account for a proportion of adults that might have spawned sapling recruits but since died. Lower per-capita sapling density around conspecific or conmycorrhizal adults than expected by the null model would indicate stronger negative CDD or CMDD, respectively. Conversely, higher per-capita sapling density around conspecific or conmycorrhizal adults (heterospecific adults of the same mycorrhizal type) than expected from the null model would indicate stronger positive CDD or CMDD, respectively.

We generated 100 iterations of the null model, using global GAMs (BAMs) (following the global model described above). We then compared our observed difference in per-capita sapling density from conspecific adults (CDD estimates) between AM and EM tree species to that derived from the 100 null model iterations. To determine this, we compared the (1) observed difference between AM and EM estimated means and (2) difference between AM and EM estimated means from each of the 100 null models, derived from GLM models testing for mycorrhizal influence on CDD (Fig. S6). This allowed us to test whether our observed stronger negative CDD in AM relative to EM tree species could be accounted for by the null model. These comparisons confirmed that observations of lower per-capita sapling density from conspecific adults for AM tree species than EM tree species could not be accounted for by mechanisms included in the null model (i.e., clumping in the adult distributions, empirically-informed dispersal limitation, and adult mortality). We repeated this null-model analysis to test for positive CMDD of AM tree species. Specifically, we used AM estimates directly instead of AM-EM differences, as our results show that CMDD is not significantly different between mycorrhizal types, but that AM tree species exhibit positive CMDD. Theses comparisons confirmed observations of greater per-capita sapling density for conmycorrhizal adults for AM tree species (Fig. S7).

### Phylogenetic signal

We checked for phylogenetic signal in per-capita sapling density metrics associated with CDD and CMDD to make sure that our species-by-site observations represented phylogenetically independent observations. We determined Pagel’s lambda for a phylogenetic tree including each unique species identified in our dataset. Pagel’s lambda is a measure of phylogenetic signal, with zero representing no signal, and one representing complete phylogenetic signal^76,77^. Species were placed on the most up to date plant backbone tree^78^. Species that were only identified to genus were added to the tree using the congeneric.merge() function in the pez package^79^. This reduced the underestimation of phylogenetic signal in the data. The low phylogenetic signal given by Pagel’s lambda for both CDD and CMDD was 0.013, which confirmed that there was little phylogenetic signal in either CDD or CMDD. Ultimately, this justified using GAM models that are not phylogenetically constrained for our second set of models testing our major hypotheses.

### Reporting summary

Further information on research design is available in the Nature Portfolio Reporting Summary linked to this article.

### Supplementary information

Supplementary Information

Reporting Summary

## Data Availability

ForestGEO plot data are not available due to data privacy and sharing restrictions, but can be obtained upon request through the ForestGEO portal: http://ctfs.si.edu/datarequest/). Other data used in the study can be found in references cited in the Methods section.

## Code availability

All code used to complete analyses for the manuscript is available at the following link: https://github.com/c383d893/Fgeo_project_Tree. Data analysis were conducted and visualizations generated in R (v. 4.2.2) and the ETH Zurich Euler cluster.
