# Supplementary material for: Mycorrhizal feedbacks influence global forest structure and diversity
Source: Commun Biol. 2023 Oct 19;6:1066. doi: 10.1038/s42003-023-05410-z (PMC10587352; doi:10.1038/s42003-023-05410-z)
Supplement: Supplementary file 1 — Supplementary Information [file 42003_2023_5410_MOESM1_ESM.pdf]

Mycorrhizal feedbacks influence global forest structure and diversity

Camille S. Delavaux[a*], Joseph A. LaManna[b], Jonathan A. Myers[c], Richard P. Phillips[d], Salomón Aguilar[e], David Allen[f], Alfonso Alonso[g], Kristina J. Anderson-Teixeira[h,e], Matthew E. Baker[i], Jennifer L. Baltzer[j], Pulchérie Bissiengou[k], Mariana Bonfim[l], Norman A. Bourg[m], Warren Y. Brockelman[n], David F.R.P. Burslem[o], Li-Wan Chang[p], Yang Chen[q], Jyh-Min Chiang[r], Chengjin Chu[s], Keith Clay[t], Susan Cordell[u], Mary Cortese[l], Jan den Ouden[v], Christopher Dick[w], Sisira Ediriweera[x], Erle C. Ellis[i], Anna Feistner[y], Amy L. Freestone[l], Thomas Giambelluca[z], Christian P. Giardina[u], Gregory S. Gilbert[aa], Fangliang He[ab], Jan Holík[ac], Robert W. Howe[ad], Walter Huaraca Huasca[ae], Stephen P. Hubbell[af], Faith Inman[ag], Patrick A. Jansen[e,v], Daniel J. Johnson[ah], Kamil Kral[ac], Andrew J. Larson[ai,aj], Creighton M. Litton[z], James A. Lutz[ak,al], Yadvinder Malhi[ae], Krista McGuire[am], Sean M. McMahon[an], William J. McShea[m], Hervé Memiaghe[am,ao], Anuttara Nathalang[n], Natalia Norden[ap], Vojtech Novotny[aq], Michael J. O'Brien[at], David A. Orwig[as], Rebecca Ostertag[ag], Geoffrey G. (‘Jess’) Parker[at], Rolando Pérez[e], Glen Reynolds[au], Sabrina E. Russo[av], Lawren Sack[af], Pavel Šamonil[ac], I-Fang Sun[aw], Mark E. Swanson[ax], Jill Thompson[ay], Maria Uriarte[az], John Vandermeer[w], Xihua Wang[ba], Ian Ware[bb], George D. Weiblen[bc], Amy Wolf[ad], Shu-Hui Wu[bd], Jess K. Zimmerman[be], Thomas Lauber[a], Daniel S. Maynard[a], Thomas W. Crowther[a], Colin Averill[a]

[a]*ETH Zurich, Department of Environmental Systems Science, Switzerland*
[b]*Department of Biological Sciences, Marquette University, Milwaukee, WI 53233, United States of America*
[c]*Department of Biology, Washington University in St. Louis, St. Louis, MO 63130, United States of America*
[d]*Department of Biology, Indiana University, Bloomington, IN 47405, United States of America*
[e]*Forest Global Earth Observatory, Smithsonian Tropical Research Institute, Panama*
[f]*Department of Biology, Middlebury College, Middlebury, VT 05753, United States of America*
[g]*Center for Conservation and Sustainability, Smithsonian Conservation Biology Institute, National Zoological Park, Washington, DC 20008, United States of America*
[h]*Forest Global Earth Observatory, Smithsonian Conservation Biology Institute, National Zoological Park, Washington, DC 20008, United States of America*
[i]*Geography & Environmental Systems, University of Maryland, Baltimore County, Baltimore, MD 21250, United States of America*
[j]*Biology Department, Wilfrid Laurier University, Waterloo, ON, N2L 3C5, Canada*
[k]*Herbier National du Gabon, Institut de Pharmacop´ee et de M´edecine Traditionelle, Gabon*
[l]*Department of Biology, Temple Ambler Field Station, Temple University, Ambler, PA 19002, United States of America*
[m]*Conservation Ecology Center, Smithsonian Conservation Biology Institute, National Zoological Park, Washington, DC 20008, United States of America*
[n]*National Biobank of Thailand, National Science and Technology Development Agency, Thailand*
[o]*School of Biological Sciences, University of Aberdeen, United Kingdom*
[p]*Taiwan Forestry Research Institute, Taipei*
[q]*State Key Laboratory of Biocontrol, School of Ecology/School of Life Sciences, Sun Yat-sen University, Guangzhou 510275, People’s Republic of China*
[r]*Department of Life Science,Tunghai University, Taiwan*

[s]*School of Life Sciences, Sun Yat-sen University, Guangzhou*
[t]*Department of Ecology and Evolutionary Biology, Tulane University, New Orleans, LA 70118, United States of America*
[u]*Institute of Pacific Islands Forestry, USDA Forest Service, Hilo, HI 96720, United States of America*
[v]*Department of Environmental Sciences, Wageningen University, Wageningen, The Netherlands*
[w]*Department of Ecology and Evolutionary Biology, University of Michigan, Ann Arbor, MI 48109, United States of America*
[x]*Department of Science and Technology, Uva Wellassa University, Sri Lanka*
[y]*Gabon Biodiversity Program, Center for Conservation and Sustainability, Smithsonian National Zoo and Conservation Biology Institute, Gamba, Gabon*
[z]*University of Hawaii at Manoa, 1910 East-West Rd., Honolulu, HI 96822*
[aa]*Environmental Studies Department, University of California, Santa Cruz, Santa Cruz, CA 95064, United States of America*
[ab]*Department of Renewable Resources, University of Alberta, Edmonton, Canada*
[ac]*Department of Forest Ecology, Silva Tarouca Research Institute, Czech Republic*
[ad]*Department of Natural and Applied Sciences, University of Wisconsin-Green Bay, Green Bay, WI 54311, United States of America*
[ae]*Environmental Change Institute, School of Geography and the Environment, University of Oxford, United Kingdom*
[af]*Department of Ecology and Evolutionary Biology, University of California, Los Angeles, Los Angeles, CA 90095, United States of America*
[ag]*Department of Biology, University of Hawaii, Hilo, HI 96720, United States of America*
[ah]*School of Forest Resources and Conservation, University of Florida, Gainesville, FL 32611, United States of America*
[ai]*Department of Forest Management, W. A. Franke College of Forestry and Conservation, University of Montana, Missoula, Montana, USA*
[aj]*The Wilderness Institute, W. A. Franke College of Forestry and Conservation, University of Montana, Missoula, Montana, USA*
[ak]*The Ecology Center, Utah State University, Logan, Utah, USA*
[al]*Wildland Resources Department, Utah State University, Logan, Utah, USA*
[am]*Department of Biology, University of Oregon, Eugene, OR 97403, United States of America*
[an]*Forest Global Earth Observatory, Smithsonian Environmental Research Center, Edgewater, MD 21037, United States of America*
[ao]*Centre National de la Recherche Scientifique et Technologique, Ouagadougou, Burkina Faso*
[ap]*Programa Ciencias de la Biodiversidad, Instituto de Investigacion ´ de Recursos Biologicos ´ Alexander von Humboldt, Colombia*
[aq]*Biology Centre, Institute of Entomology, Czech Academy of Sciences, Czech Republic*
[ar]*Universidad Rey Juan Carlos, Spain*
[as]*Harvard Forest, Harvard University, Petersham, MA 01366, United States of America*
[at]*Forest Ecology Group, Smithsonian Environmental Research Center, Edgewater, MD 21037, United States of America*
[au]*The Royal Society SEARRP (UK/Malaysia), Malaysia*
[av]*School of Biological Sciences and Center for Plant Science Innovation, University of Nebraska – Lincoln, Lincoln, NE 68588, United States of America*

[aw]*Department of Natural Resources and Environmental Studies, National Dong Hwa University, Hsinchu*
[ax]*School of the Environment, Washington State University, Pullman, Washington, USA*
[ay]*UK Centre for Ecology & Hydrology, United Kingdom*
[az]*Department of Ecology, Evolution, and Environmental Biology, Columbia University, New York, NY 10027, United States of America*
[ba]*Tiantong National Forest Ecosystem Observation and Research Station, School of Ecological and Environmental Sciences, East China Normal University, Shanghai*
[bb]*U.S. Forest Service, Institute of Pacific Islands Forestry, Pacific Southwest Research Station, Hilo, HI 96720, United States of America*
[bc]*Department of Plant & Microbial Biology, University of Minnesota, St. Paul, MN 55108, United States of America*
[bd]*Botanical Garden Division, Taiwan Forestry Research Institute, Taiwan*
[be]*Department of Environmental Sciences, University of Puerto Rico, Puerto Rico*

**camille.delavaux@usys.ethz.ch*

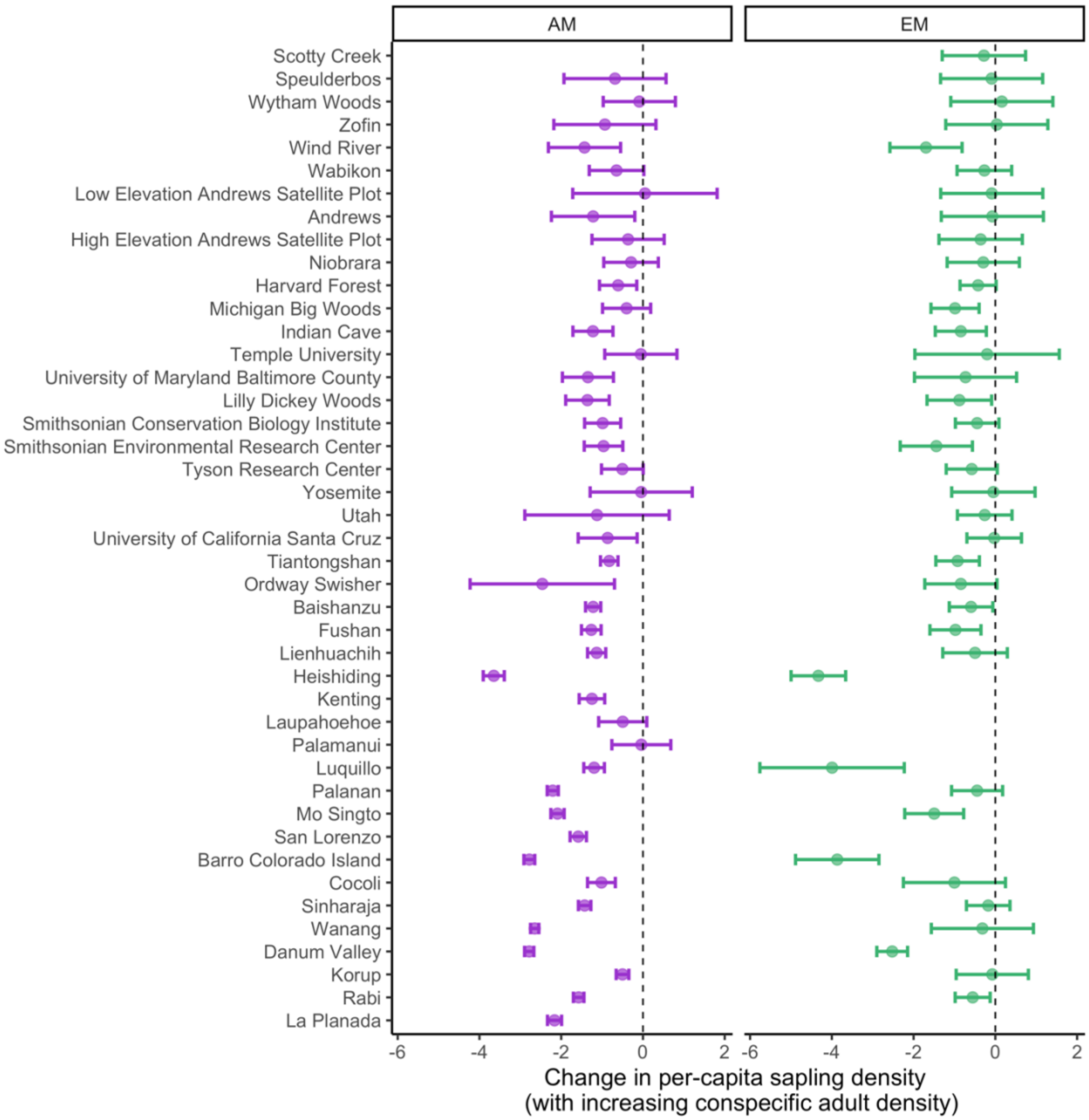

Figure S1 | Mycorrhizal type mediates strength of conspecific density dependence across sites

The effect of mycorrhizal type on conspecific density dependence, measured as estimates of change in per-capita sapling density with a standard increase in conspecific adult density (1 conspecific adult) extracted from the global model, does not vary by site, with EM tree species showing consistently weaker conspecific density dependence compared to AM tree species. Sites are ordered from high to low absolute latitude; AM and EM tree species are shown in purple and green, respectively. Error bars represent standard errors.

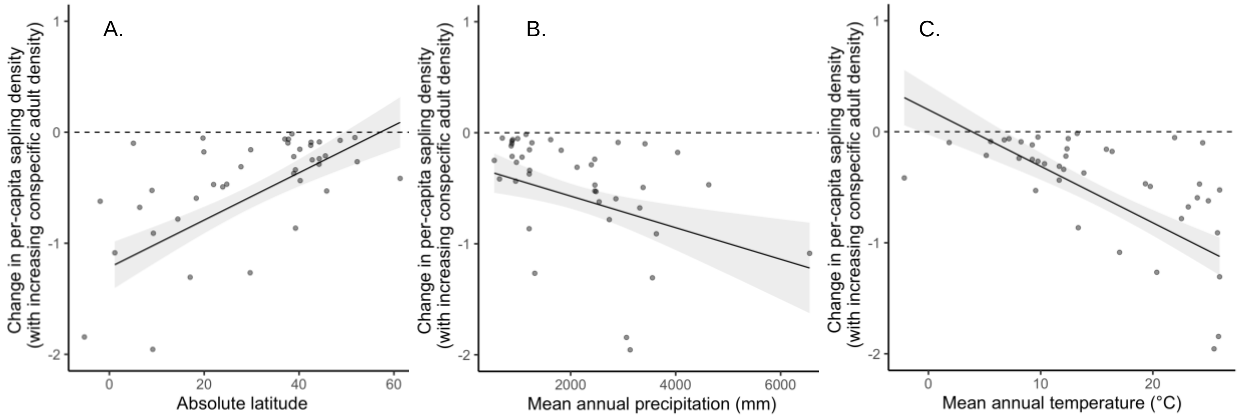

Figure S2 | Conspecific density dependence varies with absolute latitude, precipitation and temperature

The strength of conspecific density dependence is positively related to absolute latitude (A), while negatively related to mean annual precipitation (B) and temperature (C). Ribbon represents model standard errors. All values are extracted from the global model.

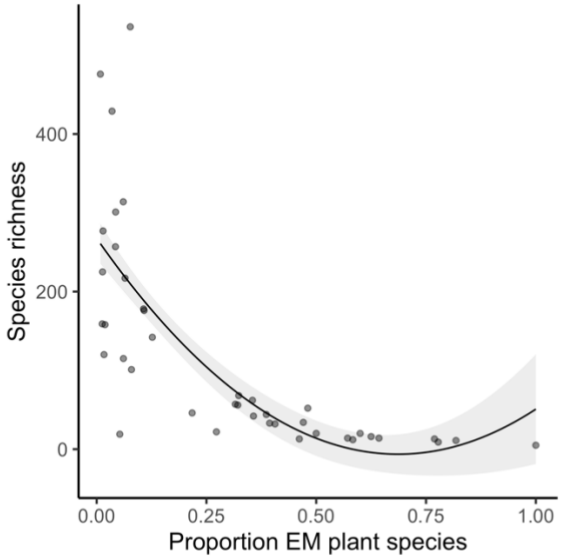

Figure S3 | Species richness is negatively related to proportion EM plant species

Species richness is negatively related to proportion ectomycorrhizal (EM) plant species. All values are extracted from the global model.

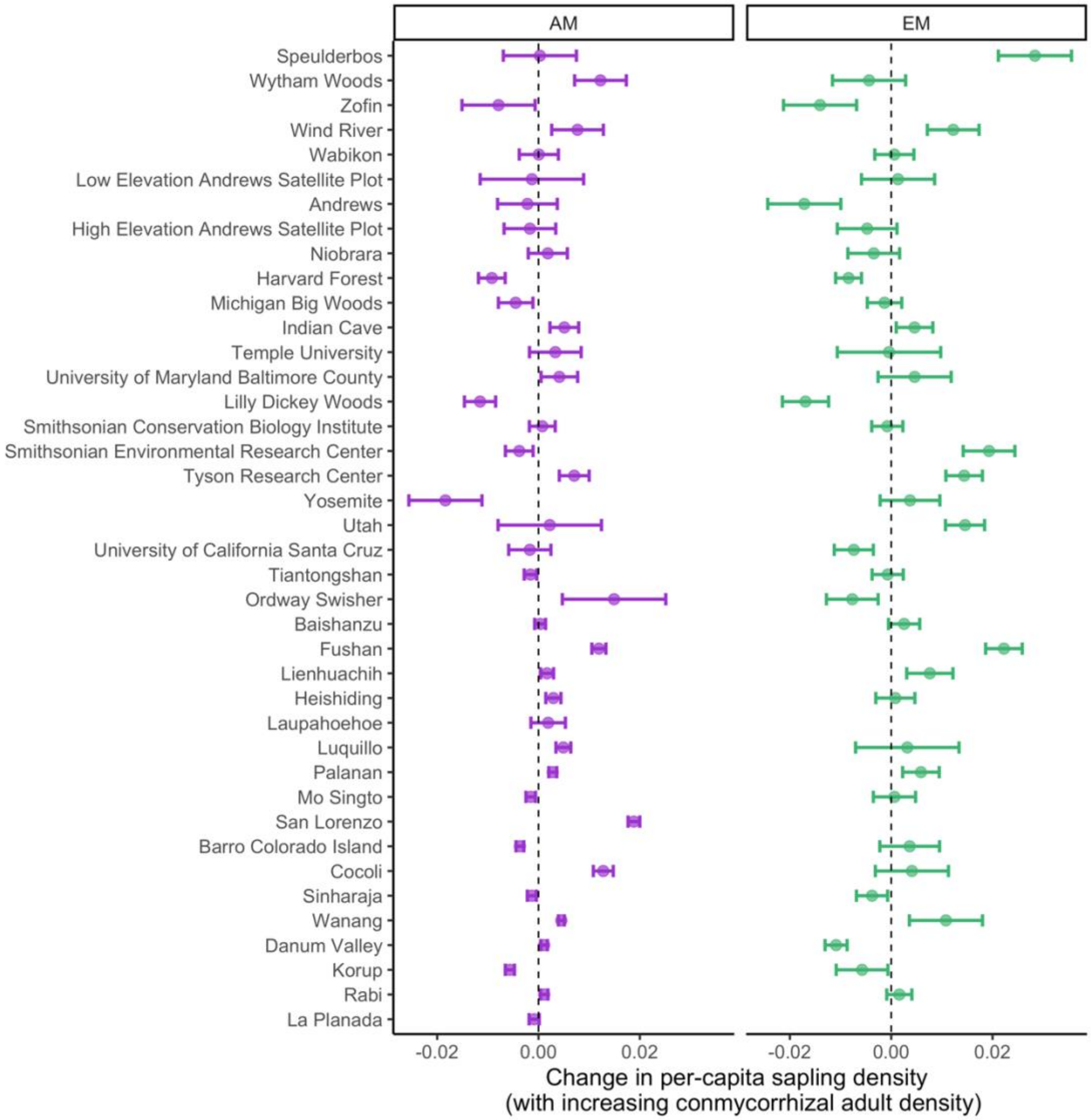

Figure S4 | Conmycorrhizal density dependence varies by site

The effect of mycorrhizal type on conmycorrhizal density dependence, measured as estimates of change in per-capita sapling density with a standard increase in conmycorrhizal adult density (1 conmycorrhizal adult) extracted from the global model, varies by site. Sites are ordered from high to low absolute latitude and include sites with sufficient AM and EM tree species for CMDD

calculation; AM and EM tree species are shown in purple and green, respectively. Error bars represent standard errors.

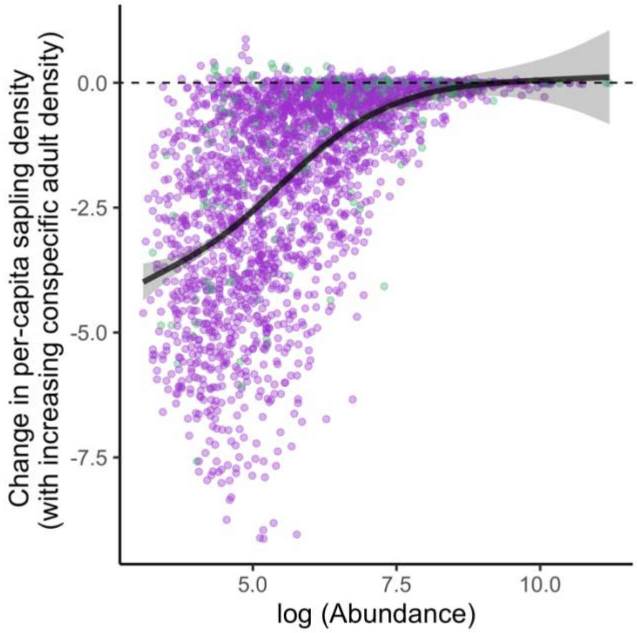

Figure S5 | Conspecific density dependence is positively related to abundance of species

Conspecific density dependence is non-linearly positively related to the natural log of species abundance, but does not vary by mycorrhizal type. AM and EM tree species are shown in purple and green, respectively; black line represents GAM model fit (R^2 = 0.335), with error bands representing 95% confidence intervals. All values are extracted from the global model.

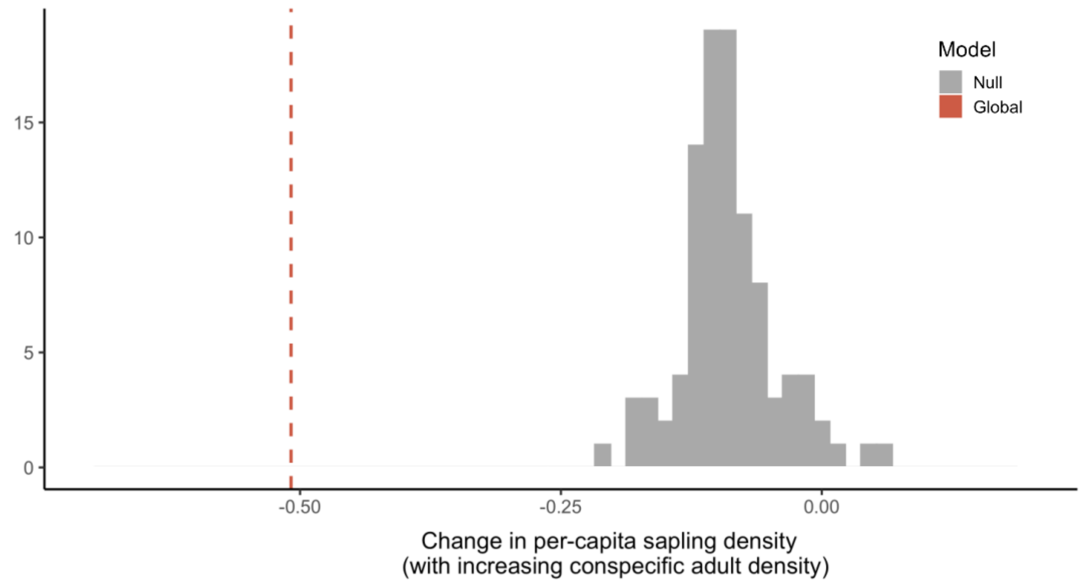

Figure S6 | Null model supports finding that mycorrhizal type mediates strength of conspecific density dependence

Observed AM-EM difference in conspecific density dependence (CDD) does not overlap the distribution of 100 null model iterations of AM-EM differences. Observed and null model iterations are shown in grey and orange, respectively. All values are extracted from the global model.

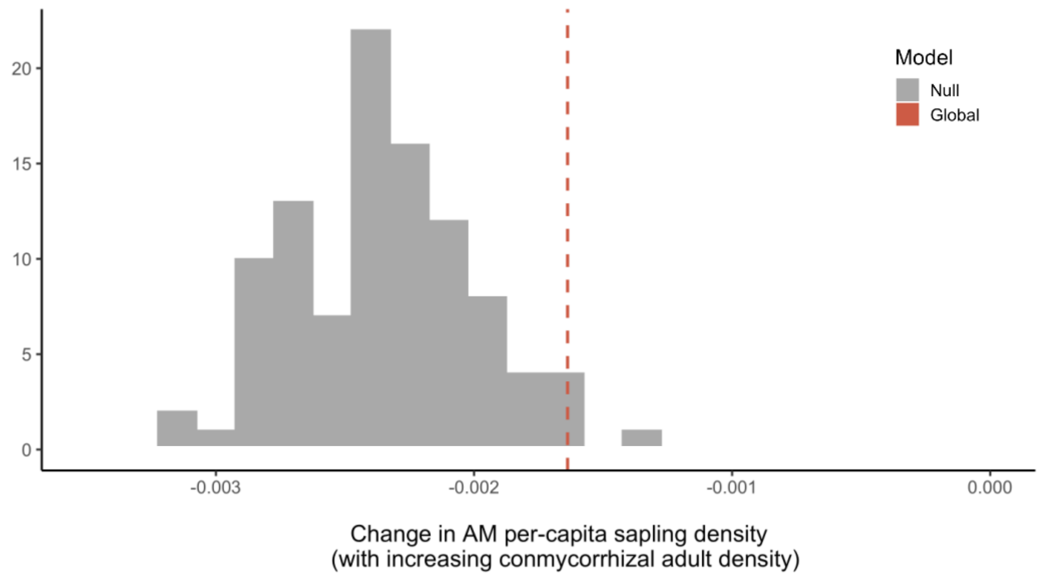

Figure S7 | Null models support finding that AM plant species experience benefit from shared mycorrhizal fungi

Observed AM conmycorrhizal density dependence (CMDD) differs significantly from the null expectation (p = 0.03). Observed and null model iterations are shown in grey and orange, respectively. All values are extracted from the global model.

Table S1 | Forest inventory plot characteristics

Basic metadata for each forest inventory plot included in the analyses, including biome (temperate, tropical or boreal, geographical coordinates (latitutde and longitude), mean annual temperature, annual precipitation, species richness and proportion species ectomycorrhizal (EM).

Forest inventory plot	Biome	Latitude	Longitude	Mean annual temperature	Annual precipitation	Species richness	Proportion EM
Baishanzu	Tropical	27.761	119.198	11.657	2121.436	178	0.107
Barro Colorado Island	Tropical	9.154	-79.846	25.457	3132.864	277	0.014

Cocoli	Tropical	8.988	-79.617	25.952	2460.069	158	0.019
Danum Valley	Tropical	5.102	117.688	25.157	2532.831	536	0.076
High Elevation Andrews Satellite Plot	Temperate	44.218	-122.243	10.343	2393.999	14	0.571
Low Elevation Andrews Satellite Plot	Temperate	44.275	-122.141	5.544	2902.765	9	0.778
Andrews	Temperate	44.261	-122.185	8.068	2457.735	14	0.643
Fushan	Tropical	24.761	121.555	19.333	4630.321	101	0.079
Harvard Forest	Temperate	42.539	-72.176	8.252	1262.944	52	0.481
Heishiding	Tropical	23.270	111.530	22.247	1441.292	176	0.108
Indian Cave	Temperate	40.247	-95.537	11.650	952.890	46	0.217
Kenting	Tropical	21.980	120.797	24.157	2461.634	80	NA
Korup	Tropical	5.074	8.855	24.442	3415.471	429	0.035
La Planada	Tropical	1.156	-77.994	17.032	6551.807	159	0.013
Laupahoehoe	Tropical	19.930	-155.287	16.367	4037.328	19	0.053
Lienhuachih	Tropical	23.914	120.879	19.772	3379.379	115	0.061
Lilly Dickey Woods	Temperate	39.236	-86.218	12.052	1218.747	33	0.394
Luquillo	Tropical	18.326	-65.816	23.962	2860.980	120	0.017
Michigan Big Woods	Temperate	42.467	-84.000	9.250	868.122	42	0.357
Mo Singto	Tropical	14.433	101.350	22.550	2735.861	257	0.043
Niobrara	Temperate	42.780	-100.021	9.253	546.285	22	0.273
Ordway Swisher	Temperate	29.691	-81.993	20.350	1316.956	11	0.818
Palamanui	Tropical	19.739	-155.994	21.940	991.757	12	NA
Palanan	Tropical	17.040	122.388	25.948	3557.002	301	0.043
Rabi	Tropical	-1.925	9.880	24.947	2542.003	314	0.061
San Lorenzo	Tropical	9.282	-79.974	25.762	3631.735	225	0.013
Smithsonian Conservation Biology Institute	Temperate	38.894	-78.145	12.247	1074.754	62	0.355

Scotty Creek	Boreal	61.300	-121.300	-2.150	645.443	5	1.000
Smithsonian Environmental Research Center	Temperate	38.889	-76.559	13.850	1211.971	68	0.324
Sinharaja	Tropical	6.402	80.402	23.158	3316.173	217	0.065
Speulderbos	Temperate	52.253	5.702	9.753	968.047	13	0.769
Temple University	Temperate	40.164	-75.192	12.347	1215.899	57	0.316
Tiantongshan	Temperate	29.812	121.783	15.853	1819.889	142	0.127
Tyson Research Center	Temperate	38.518	-90.558	13.250	1150.197	44	0.386
University of California Santa Cruz	Temperate	37.012	-122.075	12.453	897.378	32	0.406
University of Maryland Baltimore County	Temperate	39.255	-76.708	13.350	1209.918	56	0.321
Utah	Temperate	37.662	-112.853	1.848	880.484	16	0.625
Wabikon	Temperate	45.555	-88.795	5.152	885.436	34	0.471
Wanang	Tropical	-5.250	145.267	25.852	3062.931	476	0.008
Wind River	Temperate	45.820	-121.956	9.553	2482.996	20	0.600
Wytham Woods	Temperate	51.774	-1.338	9.757	699.091	20	0.500
Yosemite	Temperate	37.766	-119.819	7.173	1619.755	12	0.583
Zofin	Temperate	48.664	14.707	6.755	883.019	13	0.462

Table S2 | Model output for the global integrated model

Model outputs from the global integrated model, including a random slope and intercept for each species-by-site combination. This model includes three adult density categories (A): conspecific (A), conmycorrhizal heterospecific (CMHa) and heteromycorrhizal heterospecific

(HMHa). The CDD model includes two tree density categories (B): conspecific (A) and heterospecific (Ha).

Parametric coefficients				
R-squared, adjusted	0.352			
	Estimate	Std. Error	t value	p value
(Intercept)	-138.048	3.198	-43.166	<2.00E-16
mycEM	72.059	10.228	7.045	1.86E-12
Approximate significance of smooth terms				
	edf	Ref.df	F	p-value
s(A):mycAM	1.978	1.999	9.69E+02	<2e-16
s(A):mycEM	1.008	1.017	4.68E+01	<2e-16
s(CMHa):mycAM	1.969	1.988	7.12E+01	<2e-16
s(CMHa):mycEM	1.001	1.002	3.24E+00	0.0722
s(HMHa)	1.974	1.999	1.50E+02	<2e-16
s(sitespecies)	2149.11	2426	2.96E+06	<2e-16
s(A,sitespecies)	2288.508	2426	1.01E+04	<2e-16
s(CMHa,sitespecies)	1769.764	2426	6.55E+05	<2e-16

Table S3 | Model outputs for conspecific density dependence models

Outputs for models testing study hypotheses using data from (A) the global model and (B) species-by-site model, first treating site as a random effect, and then as a fixed effect interacting with mycorrhizal type.

A. Global Model				
Site as random effect				
R-squared, marginal	0.0060405			
R-squared, conditional	0.1567906			
	Estimate	Std. Error	t value	p value
(Intercept)	-1.3247	0.1354	-9.784	5.55E-13
mycEM	0.5086	0.1325	3.838	0.00013
Site as fixed effect				
R-squared	0.1960668			
(Intercept)	-1.21872	0.18619	-6.546	7.23E-11

mycEM	0.62462	0.56419	1.107	0.26835
siteBarro Colorado Island	-1.5612	0.22868	-6.827	1.10E-11
siteCocoli	0.20288	0.38759	0.523	0.60071
siteDanum Valley	-1.56408	0.21808	-7.172	9.82E-13
siteHigh Elevation Andrews Satellite Plot	0.85437	0.90259	0.947	0.34395
siteLow Elevation Andrews Satellite Plot	1.26718	1.77615	0.713	0.47564
siteAndrews	-0.0009	1.03667	-0.001	0.9993
siteFushan	-0.04522	0.30405	-0.149	0.88179
siteHarvard Forest	0.61095	0.49262	1.24	0.21502
siteHeishiding	-2.43103	0.31788	-7.648	2.95E-14
siteIndian Cave	-0.00518	0.52409	-0.01	0.99212
siteKenting	-0.02961	0.36355	-0.081	0.9351
siteKorup	0.71849	0.24221	2.966	0.00304
siteLa Planada	-0.94439	0.25374	-3.722	0.0002
siteLaupahoehoe	0.72366	0.61753	1.172	0.24137
siteLienhuachih	0.08588	0.29016	0.296	0.76727
siteLilley Dickey Woods	-0.13918	0.56419	-0.247	0.80517
siteLuquillo	0.02312	0.31359	0.074	0.94123
siteMichigan Big Woods	0.81844	0.61753	1.325	0.18518
siteMo Singto	-0.87214	0.24766	-3.522	0.00044
siteNiobrara	0.92722	0.6931	1.338	0.18109
siteOrdway Swisher	-1.24405	1.77615	-0.7	0.48373
sitePalamanui	1.17846	0.74476	1.582	0.11371
sitePalanan	-0.98771	0.2305	-4.285	1.90E-05
siteRabi	-0.35436	0.22641	-1.565	0.1177
siteSan Lorenzo	-0.36425	0.27325	-1.333	0.18265
siteSmithsonian Conservation Biology Institute	0.23235	0.47924	0.485	0.62785
siteScotty Creek	0.31421	1.1505	0.273	0.7848
siteSmithsonian Environmental Research Center	0.25395	0.50747	0.5	0.61683

siteSinharaja	-0.20784	0.24073	-0.863	0.38802
siteSpeulderbos	0.53476	1.26281	0.423	0.67199
siteTemple	1.16703	0.90259	1.293	0.19615
siteTiantongshan	0.39363	0.28381	1.387	0.16559
siteTyson Research Center	0.71431	0.54284	1.316	0.18834
siteUniversity of California Santa Cruz	0.35482	0.74476	0.476	0.63382
siteUniversity of Maryland Baltimore County	-0.1279	0.65167	-0.196	0.84442
siteUtah	0.09463	1.77615	0.053	0.95751
siteWabikon	0.57182	0.6931	0.825	0.40944
siteWanang	-1.42986	0.21197	-6.746	1.90E-11
siteWind River	-0.21177	0.90259	-0.235	0.81452
siteWytham Woods	1.12906	0.90259	1.251	0.21109
siteYosemite	1.17536	1.26281	0.931	0.35208
siteZofin	0.28731	1.26281	0.228	0.82004
mycEM:siteBarro Colorado Island	-1.71244	1.17301	-1.46	0.14446
mycEM:siteCocoli	-0.60974	1.41205	-0.432	0.66592
mycEM:siteDanum Valley	-0.36498	0.68776	-0.531	0.5957
mycEM:siteHigh Elevation Andrews Satellite Plot	-0.61972	1.4623	-0.424	0.67175
mycEM:siteLow Elevation Andrews Satellite Plot	-0.76048	2.2357	-0.34	0.73377
mycEM:siteAndrews	0.52189	1.70831	0.306	0.76001
mycEM:siteFushan	-0.3364	0.87527	-0.384	0.70076
mycEM:siteHarvard Forest	-0.43851	0.8493	-0.516	0.60568
mycEM:siteHeishiding	-1.30407	0.91127	-1.431	0.15255
mycEM:siteIndian Cave	-0.24528	0.97381	-0.252	0.80116
mycEM:siteKenting	NA	NA	NA	NA
mycEM:siteKorup	-0.19887	1.05939	-0.188	0.85111
mycEM:siteLa Planada	NA	NA	NA	NA
mycEM:siteLaupahoehoe	NA	NA	NA	NA
mycEM:siteLienhuachih	0.01185	0.99591	0.012	0.9905

mycEM:siteLilley Dickey Woods	-0.14733	1.10723	-0.133	0.89415
mycEM:siteLuquillo	-3.42314	1.87137	-1.829	0.06749
mycEM:siteMichigan Big Woods	-1.21035	1.00581	-1.203	0.22895
mycEM:siteMo Singto	-0.02939	0.93004	-0.032	0.9748
mycEM:siteNiobrara	-0.62849	1.24259	-0.506	0.61305
mycEM:siteOrdway Swisher	0.9951	2.05386	0.485	0.62807
mycEM:sitePalamanui	NA	NA	NA	NA
mycEM:sitePalanan	1.1356	0.85251	1.332	0.18297
mycEM:siteRabi	0.39425	0.72002	0.548	0.58405
mycEM:siteSan Lorenzo	NA	NA	NA	NA
mycEM:siteSmithsonian Conservation Biology Institute	-0.08277	0.89272	-0.093	0.92613
mycEM:siteScotty Creek	NA	NA	NA	NA
mycEM:siteSmithsonian Environmental Research Center	-1.10322	1.14942	-0.96	0.33725
mycEM:siteSinharaja	0.62794	0.79072	0.794	0.42719
mycEM:siteSpeulderbos	-0.03179	1.85428	-0.017	0.98633
mycEM:siteTemple	-0.77457	2.05386	-0.377	0.70611
mycEM:siteTiantongshan	-0.72229	0.80488	-0.897	0.3696
mycEM:siteTyson Research Center	-0.69766	0.98403	-0.709	0.4784
mycEM:siteUniversity of California Santa Cruz	0.2116	1.13315	0.187	0.85189
mycEM:siteUniversity of Maryland Baltimore County	-0.00737	1.5061	-0.005	0.9961
mycEM:siteUtah	0.23988	1.9708	0.122	0.90313
mycEM:siteWabikon	-0.24574	1.09988	-0.223	0.82322
mycEM:siteWanang	1.70693	1.37426	1.242	0.21433
mycEM:siteWind River	-0.89025	1.37052	-0.65	0.51603
mycEM:siteWytham Woods	-0.37634	1.63044	-0.231	0.81748
mycEM:siteYosemite	-0.62919	1.70831	-0.368	0.71268
mycEM:siteZofin	0.34267	1.85428	0.185	0.8534
B. Species-by-site models				

Site as random effect				
R-squared, marginal	0.000439219			
R-squared, conditional	0.003556659			
	Estimate	Std. Error	t value	p value
(Intercept)	-2.3622	0.286	-8.259	6.11E-07
mycEM	0.7771	0.8615	0.902	0.367
Site as fixed effect				
R-squared	0.01401733			
(Intercept)	-0.78277	1.12394	-0.696	0.48624
mycEM	-0.85809	3.96236	-0.217	0.82857
siteBarro Colorado Island	-3.62816	1.35919	-2.669	0.00767
siteDanum Valley	-1.43478	1.29647	-1.107	0.26858
siteHigh Elevation Andrews Satellite Plot	-0.0171	5.91183	-0.003	0.99769
siteAndrews	-0.77751	5.91183	-0.132	0.89538
siteFushan	-0.86439	1.78046	-0.485	0.62739
siteHarvard Forest	-0.35136	3.11205	-0.113	0.91012
siteIndian Cave	2.78405	3.11205	0.895	0.37112
siteKenting	-0.96599	2.23745	-0.432	0.66598
siteLa Planada	-1.78143	1.51131	-1.179	0.23866
siteLaupahoehoe	-0.45092	4.63413	-0.097	0.9225
siteLienhuachih	-0.70924	1.70879	-0.415	0.67815
siteLilley Dickey Woods	-0.23104	3.37182	-0.069	0.94538
siteLuquillo	-1.04724	1.84755	-0.567	0.5709
siteMichigan Big Woods	0.20294	3.72769	0.054	0.95659
siteMo Singto	-3.82947	1.46887	-2.607	0.00921
sitePalamanui	0.73224	5.91183	0.124	0.90144

sitePalanan	-1.03698	1.36822	-0.758	0.4486
siteRabi	-2.11674	2.26939	-0.933	0.35108
siteSan Lorenzo	-1.19507	1.59965	-0.747	0.45511
siteSmithsonian Conservation Biology Institute	-0.65835	2.91235	-0.226	0.82118
siteScotty Creek	1.15068	8.06019	0.143	0.88649
siteSinharaja	-1.69616	1.44161	-1.177	0.23952
siteTiantongshan	-0.78497	1.71685	-0.457	0.64757
siteTyson Research Center	0.41347	3.23272	0.128	0.89824
siteWabikon	0.10296	4.25517	0.024	0.9807
siteWanang	-2.13216	1.26451	-1.686	0.09194
siteWytham Woods	0.37633	10.1155	0.037	0.97033
siteZofin	-1.01869	7.19673	-0.142	0.88745
mycEM:siteBarro Colorado Island	2.81194	7.06901	0.398	0.69084
mycEM:siteDanum Valley	-2.19907	4.55099	-0.483	0.62901
mycEM:siteHigh Elevation Andrews Satellite Plot	-0.11414	12.2656	-0.009	0.99258
mycEM:siteAndrews	NA	NA	NA	NA
mycEM:siteFushan	1.04614	5.49904	0.19	0.84914
mycEM:siteHarvard Forest	1.53963	5.51708	0.279	0.78023
mycEM:siteIndian Cave	-2.06801	6.06253	-0.341	0.73306
mycEM:siteKenting	NA	NA	NA	NA
mycEM:siteLa Planada	NA	NA	NA	NA
mycEM:siteLaupahoehoe	NA	NA	NA	NA
mycEM:siteLienhuachih	1.04944	6.12935	0.171	0.86407
mycEM:siteLilley Dickey Woods	1.61598	6.78366	0.238	0.81174
mycEM:siteLuquillo	-3.073	10.9046	-0.282	0.77812
mycEM:siteMichigan Big Woods	-0.00158	6.2898	0	0.9998
mycEM:siteMo Singto	3.82129	5.78255	0.661	0.5088
mycEM:sitePalamanui	NA	NA	NA	NA
mycEM:sitePalanan	2.13924	5.37973	0.398	0.69094
mycEM:siteRabi	NA	NA	NA	NA

mycEM:siteSan Lorenzo	NA	NA	NA	NA
mycEM:siteSmithsonian Conservation Biology Institute	1.62768	5.66622	0.287	0.77395
mycEM:siteScotty Creek	NA	NA	NA	NA
mycEM:siteSinharaja	2.77663	5.56348	0.499	0.61778
mycEM:siteTiantongshan	1.52495	5.15479	0.296	0.76739
mycEM:siteTyson Research Center	0.71752	6.12535	0.117	0.90676
mycEM:siteWabikon	1.22396	7.02757	0.174	0.86175
mycEM:siteWanang	3.5379	8.15878	0.434	0.66461
mycEM:siteWytham Woods	1.48367	12.934	0.115	0.90869
mycEM:siteZofin	2.66558	12.934	0.206	0.83674

Table S4 | Model outputs for conmycorrhizal density dependence models

Outputs for models testing study hypotheses using data from (A) the global model and (B) species-by-site models, first treating site as a random effect, and then as a fixed effect interacting with mycorrhizal type.

A. Global Model				
Site as random effect				
R-squared, marginal	2.8678E-05			
R-squared, conditional	0.2721654			
	Estimate	Std. Error	t value	p value
(Intercept)	1.61E-03	1.10E-03	0.152	0.152
mycEM	-2.21E-04	7.89E-04	0.78	0.78
Site as fixed effect				
R-squared	0.2352404			
(Intercept)	3.17E-04	1.08E-03	0.295	0.768117
mycEM	2.23E-03	3.26E-03	0.683	0.494372
siteBarro Colorado Island	-3.99E-03	1.32E-03	-3.018	0.002573
siteCocoli	1.25E-02	2.24E-03	5.567	2.88E-08
siteDanum Valley	7.90E-04	1.26E-03	0.627	0.530455
siteHigh Elevation Andrews Satellite Plot	-2.02E-03	5.21E-03	-0.387	0.698988
siteLow Elevation Andrews Satellite Plot	-1.62E-03	1.03E-02	-0.158	0.874371
siteAndrews	-2.50E-03	5.99E-03	-0.418	0.675932
siteFushan	1.16E-02	1.76E-03	6.602	5.01E-11
siteHarvard Forest	-9.53E-03	2.85E-03	-3.349	0.000823
siteHeishiding	2.61E-03	1.84E-03	1.422	0.155164
siteIndian Cave	4.77E-03	3.03E-03	1.576	0.115058
siteKorup	-5.97E-03	1.40E-03	-4.264	2.09E-05
siteLa Planada	-1.18E-03	1.47E-03	-0.804	0.42176
siteLaupahoehoe	1.59E-03	3.57E-03	0.444	0.656799
siteLienhuachih	1.37E-03	1.68E-03	0.816	0.414869

siteLilley Dickey Woods	-1.19E-02	3.26E-03	-3.639	0.00028
siteLuquillo	4.58E-03	1.81E-03	2.53	0.011468
siteMichigan Big Woods	-4.83E-03	3.57E-03	-1.353	0.176077
siteMo Singto	-1.86E-03	1.43E-03	-1.298	0.194455
siteNiobrara	1.52E-03	4.00E-03	0.38	0.703939
siteOrdway Swisher	1.46E-02	1.03E-02	1.422	0.155042
sitePalanan	2.49E-03	1.33E-03	1.871	0.061473
siteRabi	8.06E-04	1.31E-03	0.616	0.537957
siteSan Lorenzo	1.85E-02	1.58E-03	11.73	<2.00E-16
siteSmithsonian Conservation Biology Institute	4.31E-04	2.77E-03	0.156	0.876407
siteSmithsonian Environmental Research Center	-4.12E-03	2.93E-03	-1.406	0.159996
siteSinharaja	-1.64E-03	1.39E-03	-1.178	0.238831
siteSpeulderbos	-4.97E-05	7.30E-03	-0.007	0.994567
siteTemple	3.00E-03	5.21E-03	0.576	0.564778
siteTiantongshan	-1.91E-03	1.64E-03	-1.163	0.245124
siteTyson Research Center	6.73E-03	3.14E-03	2.147	0.031929
siteUniversity of California Santa Cruz	-2.04E-03	4.30E-03	-0.473	0.635912
siteUniversity of Maryland Baltimore County	3.80E-03	3.77E-03	1.008	0.313502
siteUtah	1.91E-03	1.03E-02	0.186	0.852653
siteWabikon	-2.57E-04	4.00E-03	-0.064	0.948919
siteWanang	4.19E-03	1.22E-03	3.425	0.000625
siteWind River	7.38E-03	5.21E-03	1.416	0.156834
siteWytham Woods	1.19E-02	5.21E-03	2.285	0.022428

siteYosemite	-1.87E-02	7.30E-03	-2.562	0.010456
siteZofin	-8.20E-03	7.30E-03	-1.125	0.260911
mycEM:siteBarro Colorado Island	5.08E-03	6.78E-03	0.75	0.453159
mycEM:siteCocoli	-1.09E-02	8.16E-03	-1.339	0.180604
mycEM:siteDanum Valley	-1.42E-02	3.97E-03	-3.578	0.000353
mycEM:siteHigh Elevation Andrews Satellite Plot	-5.28E-03	8.45E-03	-0.624	0.532394
mycEM:siteLow Elevation Andrews Satellite Plot	4.24E-04	1.29E-02	0.033	0.973797
mycEM:siteAndrews	-1.72E-02	9.87E-03	-1.743	0.081491
mycEM:siteFushan	8.11E-03	5.06E-03	1.604	0.108928
mycEM:siteHarvard Forest	-1.43E-03	4.91E-03	-0.291	0.771161
mycEM:siteHeishiding	-4.34E-03	5.26E-03	-0.824	0.409894
mycEM:siteIndian Cave	-2.71E-03	5.63E-03	-0.483	0.629492
mycEM:siteKorup	-2.33E-03	6.12E-03	-0.381	0.703517
mycEM:siteLa Planada	NA	NA	NA	NA
mycEM:siteLaupahoehoe	NA	NA	NA	NA
mycEM:siteLienhuachih	3.71E-03	5.75E-03	0.644	0.519498
mycEM:siteLilley Dickey Woods	-7.60E-03	6.40E-03	-1.188	0.234841
mycEM:siteLuquillo	-3.95E-03	1.08E-02	-0.365	0.714823
mycEM:siteMichigan Big Woods	9.66E-04	5.81E-03	0.166	0.868033
mycEM:siteMo Singto	-4.59E-05	5.37E-03	-0.009	0.993184
mycEM:siteNiobrara	-7.52E-03	7.18E-03	-1.047	0.295183
mycEM:siteOrdway Swisher	-2.48E-02	1.19E-02	-2.092	0.036528
mycEM:sitePalanan	8.31E-04	4.93E-03	0.169	0.866007

mycEM:siteRabi	-1.76E-03	4.16E-03	-0.422	0.672882
mycEM:siteSan Lorenzo	NA	NA	NA	NA
mycEM:siteSmithsonian Conservation Biology Institute	-3.75E-03	5.16E-03	-0.727	0.467285
mycEM:siteSmithsonian Environmental Research Center	2.09E-02	6.64E-03	3.145	0.001682
mycEM:siteSinharaja	-4.69E-03	4.57E-03	-1.027	0.304303
mycEM:siteSpeulderbos	2.59E-02	1.07E-02	2.416	0.015787
mycEM:siteTemple	-5.99E-03	1.19E-02	-0.505	0.613898
mycEM:siteTiantongshan	-1.34E-03	4.65E-03	-0.288	0.773373
mycEM:siteTyson Research Center	5.12E-03	5.69E-03	0.901	0.3677
mycEM:siteUniversity of California Santa Cruz	-7.89E-03	6.55E-03	-1.206	0.228079
mycEM:siteUniversity of Maryland Baltimore County	-1.72E-03	8.70E-03	-0.198	0.843408
mycEM:siteUtah	1.01E-02	1.14E-02	0.889	0.374008
mycEM:siteWabikon	-1.67E-03	6.35E-03	-0.263	0.792656
mycEM:siteWanang	4.06E-03	7.94E-03	0.512	0.608728
mycEM:siteWind River	2.31E-03	7.92E-03	0.292	0.77049
mycEM:siteWytham Woods	-1.88E-02	9.42E-03	-1.999	0.04571
mycEM:siteYosemite	1.99E-02	9.87E-03	2.012	0.044359
mycEM:siteZofin	-8.39E-03	1.07E-02	-0.783	0.433519
B. Species-by-site models				
Site as random effect				
R-squared, marginal	0.000511088			
R-squared, conditional	0.02649235			
	Estimate	Std. Error	t value	p value
(Intercept)	0.07541	0.09401	0.802	0.434
mycEM	-0.15046	0.18038	-0.834	0.404

Site as fixed effect				
R-squared	0.04609303			
(Intercept)	0.21399	0.13104	1.633	0.102703
mycEM	-0.04923	0.954	-0.052	0.958852
siteDanum Valley	-0.30312	0.16903	-1.793	0.073153
siteAndrews	-0.1542	1.16472	-0.132	0.894694
siteFushan	-0.14806	0.3136	-0.472	0.636919
siteIndian Cave	-0.95477	1.16472	-0.82	0.412508
siteLienhuachih	-0.23866	0.27015	-0.883	0.377158
siteLilley Dickey Woods	-0.39566	0.63234	-0.626	0.531617
siteMichigan Big Woods	-1.18926	0.82878	-1.435	0.151531
siteMo Singto	-0.22832	0.21231	-1.075	0.282383
sitePalanan	-0.26788	0.19921	-1.345	0.178937
siteRabi	-0.20707	0.18685	-1.108	0.267971
siteSmithsonian Conservation Biology Institute	-0.44176	0.74359	-0.594	0.552557
siteSinharaja	-0.21162	0.20066	-1.055	0.291784
siteTiantongshan	-0.22867	0.264	-0.866	0.386547
siteTyson Research Center	-0.24043	0.82878	-0.29	0.771786
siteWanang	0.63778	0.16505	3.864	0.000117
siteWytham Woods	-0.21665	1.88991	-0.115	0.908752
siteZofin	-0.74535	1.16472	-0.64	0.52232
mycEM:siteDanum Valley	-0.1636	1.02424	-0.16	0.873116
mycEM:siteAndrews	NA	NA	NA	NA
mycEM:siteFushan	0.12545	1.19906	0.105	0.91669
mycEM:siteIndian Cave	0.6928	1.89444	0.366	0.714647
mycEM:siteLienhuachih	-0.02653	1.22543	-0.022	0.982728
mycEM:siteLilley Dickey Woods	-0.13859	1.62241	-0.085	0.931937

mycEM:siteMichigan Big Woods	0.74668	1.4545	0.513	0.607784
mycEM:siteMo Singto	-0.2372	1.50911	-0.157	0.875128
mycEM:sitePalanan	-0.09898	1.26583	-0.078	0.937686
mycEM:siteRabi	0.04168	1.05792	0.039	0.968576
mycEM:siteSmithsonian Conservation Biology Institute	0.09543	1.33444	0.072	0.943002
mycEM:siteSinharaja	0.05764	1.12608	0.051	0.959182
mycEM:siteTiantongshan	-0.01708	1.13907	-0.015	0.988036
mycEM:siteTyson Research Center	NA	NA	NA	NA
mycEM:siteWanang	-0.90214	1.50319	-0.6	0.548507
mycEM:siteWytham Woods	NA	NA	NA	NA
mycEM:siteZofin	NA	NA	NA	NA

Table S5 | Site specific acknowledgements

We thank everyone involved in the collection of the vast quantity of data in the ForestGEO network. This table details acknowledgements for each plot included in the present study.

Plot	Census	Acknowledgements
Barro Colorado Island	8	The BCI forest dynamics research project was founded by S.P. Hubbell and R.B. Foster and is now managed by R. Condit, S. Lao, and R. Perez under the Center for Tropical Forest Science and the Smithsonian Tropical Research Institute in Panama. Numerous organizations have provided funding, principally the U.S. National Science Foundation, and hundreds of field workers have contributed.[80]
Baishanzu	3	
Cocoli	3	
Danum Valley	2	The Danum plot is a core project of the Southeast Asia Rain Forest Research Partnership (SEARRP). We thank SEARRP partners, especially Yayasan Sabah for their support, and HSBC Malaysia and the University of Zurich for funding. We are grateful to the research assistants who conduct the censuses, in particular the team leader Alex Karolus, and to Mike Bernados and Bill McDonald for species identifications. We thank Stuart Davies and Shameema Esufali for advice and training.
Andrews Forest	1	We would like to acknowledge funding from the Andrews LTER site, Marquette University, and support from Dave Bell, Jeff Diez, Rob Pabst, Dave Shaw, Matt Betts, and the many technicians and

Dynamics Plots		students who helped make the Andrews Forest Dynamics Plots possible (https://forestgeo.si.edu/sites/usa/andrews). This material is based upon work supported by the National Science Foundation under the grant LTER8 DEB-2025755. Facilities were provided by the H.J. Andrews Experimental Forest and Long Term Ecological Research (LTER) program, administered cooperatively by Oregon State University, the USDA Forest Service Pacific Northwest Research Station, and the Willamette National Forest.
Fushan	2	Taiwan Forestry Bureau, Taiwan Forestry Research Institute, Tunghai University (Taiwan), Institute of Ecology and Evolutionary Biology, National Taiwan University, and the Center for Tropical Forest Science of the Smithsonian Tropical Research Institute. (USA).
Harvard Forest	1	Funding for the Harvard ForestGEO Forest Dynamics plot was provided by the Center for Tropical Forest Science and Smithsonian Institute's Forest Global Earth Observatory (CTFS-ForestGEO), the National Science Foundation's LTER program (DEB 06-20443 and DEB 12-37491) and Harvard University. Thanks to many field technicians who helped census the plot and Jason Aylward for field supervision, data screening and database management. Thanks to John Wisnewski and the woods crew for providing materials, supplies, and invaluable field assistance with plot logistics and to David Foster for his support and assistance with plot design, location, and integration with other long-term studies at HF.
Heishiding	2	National Natural Science Foundation of China (31925027) and Sun Yat-sen University.
Indian Cave	1	The Indian Cave Forest Plot is a collaborative project of the University of Nebraska-Lincoln, USA, and the Nebraska Game and Parks Commission with the Smithsonian ForestGEO program. Funding for the Indian Cave Forest Plot was provided by Jacqueline B. Mars, the Smithsonian ForestGEO program, and the University of Nebraska-Lincoln. Thanks to many field research assistants who helped census the plot and to Jess Shue who has helped with field protocols and logistics.[81]
Kenting	3	
Korup	2	The 50-ha is a collaborative project of the University of Buea, Cameroon, and the World Wide Fund for Nature, Cameroon Program in partnership with the Center for Tropical Forest Science of the Smithsonian Tropical Research Institute. Funding for the first census was provided by the International Cooperative Biodiversity Group (a consortium of the NIH, the NSF, and the USDA), with supplemental funding by the Central Africa Regional Program for the Environment (a program of USAID). Funding for the second census was provided by the Frank Levinson Family Foundation. Permission to conduct the field program in Cameroon is provided by

		the Ministry of Environment and Forests and the Ministry of Scientific Research and Innovation.
La Planada	2	The 25-ha plot La Planada Dynamics Plot is a collaborative project between the Insituto de Investigación de Recursos Biológicos Alexander von Humboldt in Colombia (Alexander von Humboldt Institute) and the Center for Tropical Forest Science of the Smithsonian Tropical Research Institute. Funding for the first census was provided by the Alexander von Humboldt Institute and funding for the second census was funded by the Smithsonian Tropical Research Institute though the Andrew W. Mellon Foundation. See Vallejo et al. (2004) for more details.
Laupāhoehoe and Pālamanui	2	The Hawaiʻi Permanent Plot Network thanks the USFS Institute of Pacific Islands Forestry (IPIF) and the Hawaiʻi Division of Forestry and Wildlife/Department of Land and Natural Resources for permission to conduct research within the Hawaiʻi Experimental Tropical Forest; the Palāmanui Group, especially Roger Harris, for access to the lowland dry forest site. We thank the Smithsonian Tropical Research Institute Center for Tropical Forest Science. This work is possible because of support provided by NSF EPSCoR (Grant Numbers EPS- 0554657 and EPS-0903833), the USDA Forest Service, the Pacific Southwest Research Station of the USFS, the University of Hawaiʻi, and the University of California at Los Angeles. We thank the USDA Forest Service and State of Hawaiʻi Department of Land and Natural Resources Division of Forestry and Wildlife for access to the Hawaiʻi Experimental Tropical Forest.
Lienhuachih	1	Taiwan Forestry Research Institute, Taiwan Forestry Bureau, Taiwan Academy of Ecology, Tunghai University (Taiwan), and the Center for Tropical Forest Science of the Smithsonian Tropical Research Institute.
Lilly Dickey Woods	1	Funding for the Lilly Dickey Woods Forest Dynamics Plot was provided by the Indiana Academy of Sciences, Indiana University Research and Teaching Preserve, and the Smithsonian Institution's Center for Tropical Forest Science.
Luquillo	6	This research was supported by grants BSR-8811902, DEB 9411973, DEB 0080538, DEB 0218039, DEB 0620910, DEB 0963447 and DEB-129764 from NSF to the Department of Environmental Science, University of Puerto Rico, and to the International Institute of Tropical Forestry USDA Forest Service, as part of the Luquillo Long-Term Ecological Research Program. The Andrew Mellon Foundation, U.S. Forest Service (Dept. of Agriculture), the University of Puerto Rico and ForestGeo gave additional support.
Michigan Big Woods	3	Thanks to the many individuals who helped census the Michigan Big Woods plot. Plot censuses were funded by the Edwin S. George Reserve Fund of the Department of Ecology and Evolutionary

		Biology at the University of Michigan and by a USDA McIntyre-Stennis Grant. We thank Stuart Davies and the Smithsonian staff for guidance in establishing a ForestGEO plot.
Mo Singto	4	Thai National Park, Wildlife and Plant Conservation Department; Thai Ministry of Natural Resources and Environment; National Center for Genetic Engineering and Biotechnology (Thailand); National Science and Technology Development Agency (Thailand).
Niobrara	1	The Niobrara Forest Plot is a collaborative project of the University of Nebraska-Lincoln, USA, and The Nature Conservancy's Niobrara Valley Preserve with the Smithsonian ForestGEO program. Funding for the Niobrara Forest Plot was provided by Jacqueline B. Mars, the Smithsonian ForestGEO program, and the University of Nebraska-Lincoln. Thanks to the many field research assistants who have helped census the plot and to Jess Shue who has helped with field protocols and logistics.[82]
Ordway Swisher	1	The establishment of the Ordway Swisher Forest Dynamics Plot was made possible by an Ordway Swisher Biological Station Jumpstart grant from UF Institute for Food and Agriculture.
Palanan	4	Isabela State University (Philippines), Conservation International, PLAN, Arnold Arboretum of Harvard University (USA).
Rabi	1	The Rabi 25-ha is a collaborative project of the National Center for Scientific and Technical Research (CENAREST) in Gabon, the Center for Conservation and Sustainability (CCS) of the Smithsonian National Zoo and Conservation Biology Institute (NZCBI) and the Center for Tropical Forest Science - Forest Global Earth Observatories (CTFS-ForestGEO) of the Smithsonian Tropical Research Institute (STRI). Funding for the first census was provided by Shell Gabon, CTFS-ForestGEO, and Smithsonian NZCBI. Permission to conduct the field program in Gabon is provided by CENAREST. The plot is located in a conservation area of overlapping oil (Assala Gabon) and forestry concessions (Compagnie des Bois du Gabon (CBG)). Samples were collected by Gauthier Moussavou and Landry Tchignoumba under permit AR037/22//MESRSTTCA/CENAREST/CG/CST/CSAR.
Smithsonian Conservation Biology Institute	3	Funding for the Smithsonian Conservation Biology Institute (SCBI) Large Forest Dynamics Plot (LFDP) was provided by the Smithsonian Institution, the National Zoological Park, and the HSBC Climate Partnership. The SCBI LFDP is part of the Smithsonian Institution Forest Global Earth Observatory, a worldwide network of large, long-term forest dynamics plots.
Smithsonian Environmental Research Center	2	Smithsonian Environmental Research Center, Earthwatch Institute
Sinharaja	3	The 25-ha Long-Term Ecological Research Project at Sinharaja World Heritage Site is a collaborative project of the University of

		Peradeniya, the Center for Tropical Forest Science of the Smithsonian Tropical Research Institute and the Arnold Arboretum of Harvard University, USA, with supplementary funding received from the John D. and Catherine T. Macarthur Foundation, the National Institute for Environmental Science, Japan, and the Helmholtz Centre for Environmental Research-UFZ, Germany, for past censuses. The PIs gratefully acknowledge the Forest Department and the Post-Graduate Institute of Science at the University of Peradeniya, Sri Lanka for supporting this project, and the local field and lab staff who tirelessly contributed in the repeated censuses of this plot.
San Lorenzo	4	
Scotty Creek	2	The authors thank R. Patankar, C. Wallace, K. Dearborn and many field assistants for their contributions in establishing the Scotty Creek ForestGEO<br>plot. We would like to thank the Dehcho First Nation for permission to conduct research on their territory, and the Scotty Creek Research Station for providing accommodations during fieldwork (Aurora Research Institute Scientific Research License number 16431). We are grateful for financial support from the following agencies: Natural Science and Engineering Research Council of Canada, Canada Foundation for Innovation, Canada Research Chairs, Canadian Foundation for Climate and Atmospheric Science, Smithsonian ForestGEO program, the CFREF Global Water Futures project Northern Water Futures, and the Northern Scientific Training Program. The Government of the Northwest Territories – Wilfrid Laurier University Partnership provided important logistical support.
Speulderbos	1	The ForestGeo site Speulderbos is maintained by Wageningen University. We thank the many staff members and students that assisted in collecting the field data, and special thanks to the Geo-information and Remote sensing group for georeferencing the plot. Also thanks to the State Forest Service for granting access to the site, and STRI for their help in establishing the plot.
Temple University		The Temple Forest Observatory is supported by the Temple Ambler Field Station at Temple University and was established in collaboration with the Smithsonian ForestGEO Network. We thank the many undergraduate student interns who assisted with data collection and the staff at Temple Ambler Campus for logistical support.
Tiantongshan	2	

Tyson Research Center	1	Funding for the Tyson Research Center ForestGEO Plot was provided by the International Center for Advanced Renewable Energy and Sustainability (I-CARES) at Washington University in St. Louis, ForestGEO, the National Science Foundation (DEB 1557094, DEB 2240431), Tyson Research Center, and Washington University in St. Louis' Provost's Office. We thank the Tyson Research Center staff and the more than 140 research technicians and students that have contributed to the project.
University of California Santa Cruz	3	The UCSC Forest Ecology Research Plot was made possible by National Science Foundation grants to Gregory S. Gilbert (DEB-0515520, DEB-084259, and DEB-1655896), by the Pepper-Giberson Chair Fund, the Robert Headley Presidential Chair for Integral Ecology and Environmental Justice, the UCSC Campus Natural Reserve, the University of California, the ForestGEO global network, and by the hard work of hundreds of UCSC students. We acknowledge that the land on which this research was conducted is the unceded territory of the Awaswas-speaking Uypi Tribe. The Amah Mutsun Tribal Band, comprised of the descendants of indigenous people taken to missions Santa Cruz and San Juan Bautista during Spanish colonization of the Central Coast, is today working hard to restore traditional stewardship practices on these lands and heal from historical trauma.
University of Maryland Baltimore County	2	The UMBC Forest Dynamics Plot was made possible through a USDA Forest Service Joint Venture Agreement (06-JV-11242300-135) to Erle Ellis, a US National Science Foundation grant (DBI-1147089) awarded to Erle Ellis and Marc Olano, and NSF IGERT (IGERT-0549469) to Claire Welty at CUERE (UMBC's Center for Urban Environmental Research and Education). Additional support has come from UMBC to Matthew Baker. We thank the numerous graduate and undergraduate students who have contributed to data collection. UMBC was established upon the land of the Piscataway and Susquehannock peoples. Over time, citizens of many more Indigenous nations have come to reside in this region. We humbly offer our respect to all past, present, and future Indigenous people connected to this place.
Utah	2	The Utah Forest Dynamics Plot is a collaborative project of Utah State University and the Utah Agricultural Experiment Station. Funding has been provided by Utah State University, the Ecology Center at Utah State University, the Utah Agricultural Experiment Station (projects 1153, 1398, and 1423), and private donors. We thank Cedar Breaks National Monument for providing logistical support, and the students, volunteers and staff individually listed at http://ufdp.org for data collection. Research was performed under annual National Park Service research permits for study CEBR-00016.[83,84]

Wabikon Lake	3	The Wabikon Lake Forest Dynamics Plot, located in the Chequamegon-Nicolet National Forest of northern Wisconsin, is part of the Smithsonian Institution's CTFS-ForestGEO network. Tree censuses at the site have been supported by The 1923 Fund, the Smithsonian Tropical Research Institute, and the Cofrin Center for Biodiversity at the University of Wisconsin-Green Bay. More than 50 scientists and student assistants contributed to the first two plot censuses. We are particularly grateful for the leadership of Gary Fewless, Steve Dhein, Kathryn Corio, Juniper Sundance, Cindy Burtley, Curt Rollman, Mike Stiefvater, Kim McKeefry, and U.S. Forest Service collaborators Linda Parker and Steve Janke.
Wanang	1	The 50-ha Wanang Forest Dynamics Plot is a collaborative project of the New Guinea Binatang Research Center, the Center for Tropical Forest Science of the Smithsonian Tropical Research Institute, the Forest Research Institute of Papua New Guinea, the Czech Academy of Sciences (grant GACR 19-28126X) and the University of Minnesota (NSF DEB- 1027297). We acknowledge the government of Papua New Guinea and the customary landowners of Wanang for supporting and maintaining the plot.
Wind River	2	The Wind River Forest Dynamics Plot is a collaborative project of Utah State University and the USDA Forest Service Pacific Northwest Research Station. Funding has been provided by the Smithsonian ForestGEO, Utah State University, the Utah Agricultural Experiment Station (projects 1153, 1398, and 1423), the National Science Foundation (DEB #1542681), and private donors. We acknowledge the Gifford Pinchot National Forest and the U.S. Forest Service Wind River Field Station for providing logistical support, and the students, volunteers and staff individually listed at http://wfdp.org for data collection. Research was performed under long-term research permits issued by the USFS with validity from 2010 – 2040. [85,86]
Wytham Woods	3	Data collection at Wytham Woods was funded by an European Research Council Advanced Investigator Award (GEM-Traits) to YM (Grant 321131) and by UK Natural Environment Research Council (NERC) Grant NE/T007648/1.
Yosemite	1	The Yosemite Forest Dynamics Plot is a collaborative project of Utah State University, the University of Montana, and Washington State University. Funding has been provided by the Smithsonian ForestGEO, Utah State University, the Joint Fire Science Program 16-1-04-02), the National Park Service P14AC00122 and P14AC00197), the Ecology Center at Utah State University, the Utah Agricultural Experiment Station (projects 1153, 1398, and 1423), and private donors. We thank Yosemite National Park for providing logistical support, and the students, volunteers and staff individually listed at http://yfdp.org for data collection. Research

		was performed under annual National Park Service research permits for study YOSE-0051.[84,87]
Zofin	5	The Zofin Forest Dynamics Plot is part of the Smithsonian Institution Forest Global Earth Observatory, a worldwide network of large, long-term forest dynamics plots. We acknowledge the Department of Forest Ecology of the Silva Tarouca Research Institute for supporting and maintaining the long-term monitoring of the Zofin Forest Dynamics Plot under the Czech Science Foundation, grant No.23-06745S.[88-90]
