# Supplementary material for: Mycorrhizal feedbacks influence global forest structure and diversity
Source: Commun Biol. 2023 Oct 19;6:1066. doi: 10.1038/s42003-023-05410-z (PMC10587352; doi:10.1038/s42003-023-05410-z)
Supplement: Supplementary file 2 — Reporting Summary [file 42003_2023_5410_MOESM2_ESM.pdf]

nature portfolio

Corresponding author(s): Camille S. Delavaux

Last updated by author(s): 09/18/2023

Reporting Summary

Nature Portfolio wishes to improve the reproducibility of the work that we publish. This form provides structure for consistency and transparency in reporting. For further information on Nature Portfolio policies, see our Editorial Policies and the Editorial Policy Checklist.

Statistics

For all statistical analyses, confirm that the following items are present in the figure legend, table legend, main text, or Methods section.

n/a	Confirmed	
☐	☒	The exact sample size (*n*) for each experimental group/condition, given as a discrete number and unit of measurement
☐	☒	A statement on whether measurements were taken from distinct samples or whether the same sample was measured repeatedly
☐	☒	The statistical test(s) used AND whether they are one- or two-sided<br>*Only common tests should be described solely by name; describe more complex techniques in the Methods section.*
☐	☒	A description of all covariates tested
☐	☒	A description of any assumptions or corrections, such as tests of normality and adjustment for multiple comparisons
☐	☒	A full description of the statistical parameters including central tendency (e.g. means) or other basic estimates (e.g. regression coefficient) AND variation (e.g. standard deviation) or associated estimates of uncertainty (e.g. confidence intervals)
☐	☒	For null hypothesis testing, the test statistic (e.g. *F*, *t*, *r*) with confidence intervals, effect sizes, degrees of freedom and *P* value noted<br>*Give P values as exact values whenever suitable.*
☒	☐	For Bayesian analysis, information on the choice of priors and Markov chain Monte Carlo settings
☐	☒	For hierarchical and complex designs, identification of the appropriate level for tests and full reporting of outcomes
☒	☐	Estimates of effect sizes (e.g. Cohen's *d*, Pearson's *r*), indicating how they were calculated

Our web collection on statistics for biologists contains articles on many of the points above.

Software and code

Policy information about availability of computer code

Data collection	No software was used to collect data in this study.
Data analysis	All code used to complete analyses for the manuscript is available at the following link: https://github.com/c383d893/Fgeo_project_Tree.<br>Data analysis were conducted and visualizations generated in R (v. 4.2.2) and the ETH Zurich Euler cluster.

For manuscripts utilizing custom algorithms or software that are central to the research but not yet described in published literature, software must be made available to editors and reviewers. We strongly encourage code deposition in a community repository (e.g. GitHub). See the Nature Portfolio guidelines for submitting code & software for further information.

Data

Policy information about availability of data

All manuscripts must include a data availability statement. This statement should provide the following information, where applicable:

- Accession codes, unique identifiers, or web links for publicly available datasets
- A description of any restrictions on data availability
- For clinical datasets or third party data, please ensure that the statement adheres to our policy

ForestGEO plot data are not available due to data privacy and sharing restrictions, but can be obtained upon request through the ForestGEO portal: http://ctfs.si.edu/datarequest/). Other data used in the study can be found in references cited in the Methods section.

Human research participants

Policy information about studies involving human research participants and Sex and Gender in Research.

Reporting on sex and gender	N/A
Population characteristics	N/A
Recruitment	N/A
Ethics oversight	N/A

Note that full information on the approval of the study protocol must also be provided in the manuscript.

Field-specific reporting

Please select the one below that is the best fit for your research. If you are not sure, read the appropriate sections before making your selection.

☐ Life sciences ☐ Behavioural & social sciences ☒ Ecological, evolutionary & environmental sciences

For a reference copy of the document with all sections, see nature.com/documents/nr-reporting-summary-flat.pdf

Ecological, evolutionary & environmental sciences study design

All studies must disclose on these points even when the disclosure is negative.

Study description	Our analytical approach consisted of (1) modelling sapling density around nearby conspecific and heterospecific adults (per-capita sapling density, the number of saplings per quadrat divided by the number of conspecific or heterospecific adult trees per quadrat), (2) predicting the change in per-capita sapling density for a standard increase in adult density, and (3) using either models (A) directly or (B) resulting predictions in a second set of models to test our hypotheses (see sections 'Per-capita sapling density GAM models' and 'Estimating change in per capita sapling density from models' below for details). We tested for (1) systematic differences in conspecific saplings per adult (per-capita sapling density) between the two mycorrhizal types; we hypothesized that EM tree species show a higher density of conspecific saplings per adult (per-capita sapling density), as predicted under weaker negative CDD (H1). We also test for evidence of positive community level mycorrhizal feedback, or conmycorrhizal density dependence (CMDD) in both mycorrhizal types (H2).
Research sample	Our study included tree-census data from 43 sites that are part of the Forest Global Earth Observatory (ForestGEO) network (Table S1; 23 temperate, 19 tropical, 1 boreal).
Sampling strategy	Sample size was based on data availability and criteria described in data exclusions; we used all usable data.
Data collection	Each site contains a large forest plot in which all free standing woody stems (hereafter trees) > 1 cm DBH (diameter at breast height, 1.3 m from the ground) were measured, identified, and geolocated following the same protocol.
Timing and spatial scale	The most recent census was used for every site except for Yosemite, where we used the earliest census (2009-10) to avoid the confounding effect of fire-caused mortality in later censuses, and the Luquillo plot, where the census in 2011 prior to hurricane Maria (2016) was used. The unit of replication we used was the quadrat, representing 20 x 20 m subplots across each plot. We chose this unit as it is a reasonable scale at which to expect spatial patterns due to density dependence based on previous studies.
Data exclusions	We converted the data from one census from each site to a universal format, using the following variables extracted from the censuses for each individual stem: full latin name, genus, species, the x-coordinate position within the plot, the y-coordinate position within the plot, the DBH, and status (alive or dead). We kept all live trees whether they were broken or otherwise damaged to retain as much recorded data as possible; all dead stems (9.9% of stems) were excluded from downstream analyses. The most recent census was used for every site except for Yosemite, where we used the earliest census (2009-10) to avoid the confounding effect of fire-caused mortality in later censuses, and the Luquillo plot, where the census in 2011 prior to hurricane Maria6(2016) was used. This resulted in a dataset comprised of 43 sites and a total of 3,299,000 stems (2,775,129 trees, or main stems) across 4,161 species. All metadata was calculated using stem data, whereas subsequent analyses were always carried out using only main stems (the largest stem of an individual). We only analyzed species that were reported as strictly arbuscular mycorrhizal (AM) or ectomycorrhizal (EM) in this database, and removed all ambiguous categories (AMEM, AMNM, where NM is non-mycorrhizal), other mycorrhizal types, and unmatched plant species; this resulted in the removal of 6.1% of observations (stems).
Reproducibility	Findings are reproducible following analysis code (https://github.com/c383d893/Fgeo_project_Tree).
Randomization	Allocation was not random. Allocation to species was defined in data shared by each site. We determined metadata at three levels: (1) the species level, (2) the site level and (3) the species-by-site level; all metadata was calculated using the full stem dataset. At the species level (i.e. for each species), we determined species level or if not possible, putative genera-based mycorrhizal status. To do this, we relied on the FungalRoot database, first assigning to known species mycorrhizal status, and if species data was unavailable in either the census data or the FungalRoot database, to genus level mycorrhizal status.

Blinding	Blinding was not relevant here (See "Randomization").

Did the study involve field work? ☐ Yes ☒ No

Reporting for specific materials, systems and methods

We require information from authors about some types of materials, experimental systems and methods used in many studies. Here, indicate whether each material, system or method listed is relevant to your study. If you are not sure if a list item applies to your research, read the appropriate section before selecting a response.

Materials & experimental systems

n/a	Involved in the study
☒	☐ Antibodies
☒	☐ Eukaryotic cell lines
☒	☐ Palaeontology and archaeology
☒	☐ Animals and other organisms
☒	☐ Clinical data
☒	☐ Dual use research of concern

Methods

n/a	Involved in the study
☒	☐ ChIP-seq
☒	☐ Flow cytometry
☒	☐ MRI-based neuroimaging
